# Harnessing Phytonanotechnology to Tackle Neglected Parasitic Diseases: Focus on Chagas Disease and Malaria

**DOI:** 10.3390/pharmaceutics17081043

**Published:** 2025-08-12

**Authors:** Manuela García, María S. Magi, Mónica C. García

**Affiliations:** 1 Departamento de Química Orgánica, Facultad de Ciencias Químicas, Universidad Nacional de Córdoba, Science Building 2, Haya de la Torre and Medina Allende, Ciudad Universitaria, Córdoba X5000HUA, Argentina; manuelagarcia@unc.edu.ar; 2 Instituto Multidisciplinario de Biología Vegetal (IMBIV), Consejo Nacional de Investigaciones Científicas y Técnicas (CONICET), Córdoba X5000HUA, Argentina; 3 Departamento de Ciencias Farmacéuticas, Facultad de Ciencias Químicas, Universidad Nacional de Córdoba, Science Building 2, Haya de la Torre and Medina Allende, Ciudad Universitaria, Córdoba X5000HUA, Argentina; maria.sol.magi@unc.edu.ar; 4 Unidad de Investigación y Desarrollo en Tecnología Farmacéutica (UNITEFA), Consejo Nacional de Investigaciones Científicas y Técnicas (CONICET), Córdoba X5000HUA, Argentina

**Keywords:** natural products, Chagas disease, malaria, phytonanomedicine, plant-based bioactive compounds, delivery of products of natural origin

## Abstract

Neglected parasitic diseases such as Chagas disease and malaria continue to pose major public health challenges, particularly in low-resource settings. Current therapies are often limited by high toxicity, poor efficacy, drug resistance, and limited accessibility. Phytochemicals, naturally occurring compounds in plants, have played a crucial role in medicine since ancient times and have gained renewed attention for their demonstrated antiparasitic activity. However, many products of natural origin (PNOs) face significant barriers to clinical use, including poor solubility, low bioavailability, and chemical instability. These limitations have driven researchers to explore alternative and innovative approaches based on the use of PNOs to tackle these parasitic infections more effectively. This review provides a comprehensive overview of key PNOs with proven activity against *Trypanosoma cruzi* and *Plasmodium* spp., the causative agents of Chagas disease and malaria, respectively. Recent advances in the design of phytonanoformulations are analyzed and discussed, emphasizing the potential of nanocarrier-based systems incorporating PNOs as a strategy to improve the pharmacokinetic and therapeutic properties of these natural products. By critically examining the integration of phytochemicals into nanotechnology-based drug delivery platforms, this review highlights the promise of phytonanotechnology to overcome current limitations in antiparasitic therapy and support the development of more effective and accessible treatments for neglected parasitic diseases.

## 1. Introduction

Neglected diseases, also referred to as neglected tropical diseases, represent a diverse group of infectious conditions that disproportionately affect more than 2.7 billion people worldwide, primarily in low- and middle-income countries across Africa, Asia, and Latin America. Often known as diseases of poverty, they are caused by a range of pathogens, including parasitic organisms, and are associated with high rates of morbidity and mortality in endemic regions. Despite their substantial global impact, neglected diseases have historically received limited attention and funding, largely due to their being concentrated in impoverished areas, which offer little financial incentive for pharmaceutical companies to invest in drug development [1]. This situation has created an urgent need for a novel, effective, and accessible therapeutic option. In this context, natural sources, particularly plants, have emerged as a promising reservoir of bioactive products with significant antiparasitic potential.

Products of natural origin (PNOs) have been used in traditional medicine and continue to serve as an invaluable source of inspiration in modern drug design and development [2,3]. According to recent reports, PNOs and their derivatives account for nearly 50% of the 1881 new chemical entities approved as drugs by the U.S. Food and Drug Administration between 1981 and 2019 [4].

Structurally, PNOs often present advantages over synthetic compounds. They are considered privileged structures refined through evolutionary processes over millennia [5]. The clinical success of many drug candidates is closely linked to the three-dimensional nature of their molecular structures and the presence of a wide range of functional groups arranged in unique spatial configurations [6,7]. However, despite these advantages, the structural complexity of PNOs, as well as their potential toxicity and unfavorable pharmacokinetics, can limit their clinical applicability. In many cases, structural modification is necessary to improve drug-like properties [8,9].

Medicinal chemistry has successfully addressed several of these challenges through the structural modification of parent compounds. Nevertheless, analyses indicate that only approximately 20% of naturally occurring lead compounds reach the clinic without structural changes [10]. Even modified compounds frequently face challenges related to solubility, stability, or bioavailability, requiring new design and formulation strategies to optimize PNOs performance and facilitate clinical translation.

There is growing support for the exploration of PNOs (whether as extracts, mixtures, or pure compounds) as therapeutic agents for neglected diseases such as Chagas and malaria, owing to their demonstrated antiparasitic activity across various stages of the parasitic life cycle. Numerous studies have described the composition, diversity, and efficacy of extracts and herbal preparations derived from antiparasitic plants [11]. The compounds reported to date can be grouped into several structural categories, which are reviewed in the following sections. Successful examples of the derivatization of PNOs and relevant herbal preparations are also discussed. A recent review of drug discovery for Chagas disease in Latin America found that nearly one-third of studies published between 2010 and 2021 reported positive results derived from PNO, whether isolated from plants or other organisms or inspired by natural structures [12]. This finding aligns with the strong ethnobiological and ethnopharmacological traditions in the region and reflects the sustained research efforts of Latin American institutions. PNO-based therapies remain essential components of anti-infective treatment strategies. However, the discovery of new drugs from natural sources poses specific challenges, including the isolation and characterization of pure compounds and the optimization of their pharmacological properties. Despite these obstacles, the field has experienced a resurgence, fueled by advances in automation and modern *omics* technologies.

The history of antimalarial chemotherapy is closely intertwined with that of herbal medicines. Quinine, the first PNO identified for antimalarial use, was isolated from the bark of the *Cinchona* tree in 1820. Although Chagas disease and malaria share some characteristics, their treatment strategies differ due to various factors, including differences in disease evolution (acute vs. chronic), available resources, affected populations, diagnostic approaches, treatment accessibility, and drug availability [13].

While structural modification remains a valuable strategy to enhance the biological performance of PNOs [8,9], many plant-derived compounds still face challenges related to poor water solubility, low bioavailability, limited absorption, and susceptibility to degradation [14]. These limitations have prompted the exploration of alternative strategies, among which phytonanotechnology has gained considerable attention. Numerous studies have proposed the incorporation of phytochemicals into nanocarrier-based drug delivery systems as a means to improve their therapeutic performance [15]. Nanodelivery systems can significantly enhance solubility, chemical stability, and therapeutic efficacy, while modulating biodistribution profiles [16]. Furthermore, phytonanotechnology enables controlled or sustained release and may reduce systemic toxicity, thereby reinforcing its relevance in the development of modern phytopharmaceutical formulations [16,17,18].

This review provides a comprehensive overview of the literature from recent decades, with a focus on key PNOs exhibiting significant biological activity against Chagas disease and malaria. A structured and reproducible methodology was followed, using specific keywords to guide the literature search. Original research articles and reviews published since 2010 were primarily included, along with selected books and book chapters. Earlier studies were also considered when the relevance of the bioactive compounds warranted their inclusion. Special emphasis is placed on the development of PNO-based phytonanoformulations as innovative platforms to enhance the therapeutic potential of these bioactive agents. By examining the integration of phytochemicals into nanocarriers, this review aims to demonstrate how such strategies can help overcome current treatment limitations, such as low bioavailability, toxicity, and drug resistance, and contribute to more effective and accessible therapies for these persistent infections.

Importantly, this review bridges two traditionally distinct domains: the pharmacological potential of plant-derived PNOs and recent advances in nanotechnology-based drug delivery. By integrating both areas, the review offers a holistic perspective on the development of innovative therapies for neglected parasitic diseases. The discussion focuses on *Trypanosoma* and *Plasmodium* species, the causative agents of Chagas disease and malaria, respectively, and highlights how the combination of phytochemicals and nanocarriers can synergistically address existing therapeutic challenges. To enhance clarity and coherence, the manuscript is organized into three main sections: antiparasitic phytochemicals for Chagas disease, phytochemicals for malaria, and a comprehensive overview of phytonanotechnological approaches. This integrated framework aims to support translational efforts toward safer, more effective, and accessible treatments for these global health burdens.

## 2. Antichagasic PNOs

### 2.1. Alkaloids

**Figure 1:**
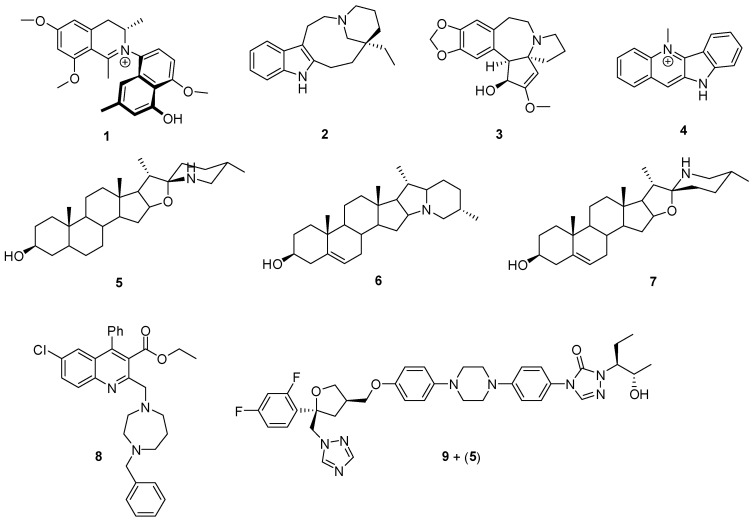
Representative alkaloids with anti-*T. cruzi* properties.

This section presents a selection of representative studies from recent decades that investigate alkaloids as trypanocidal agents from different perspectives (Figure 1).

From a phytochemical standpoint, a notable report from 2011 described the isolation of the first N-8′ coupled to naphthylisoquinoline alkaloid containing free phenolic OH groups (4′-O-demethylancistrocladinium A) from the leaves and bark of *Ancistrocladus cochinchinensis*. This species also contains ancistrocladinium A (a nonphenolic alkaloid) and four compounds featuring C-C coupling. The monophenolic alkaloid [4′-O-demethylancistrocladinium A (**1**)] demonstrated potent trypanocidal activity, surpassing benznidazole (reference drug and the first-line treatment for Chagas disease) in both the half-maximal inhibitory concentration (IC_50_ = 0.03 mM) and selectivity index (SI = 79.1) against *T. cruzi* amastigotes [19].

From a bioinformatic perspective, molecular docking and binding mode analyses have been employed to evaluate alkaloid interactions with parasite target proteins. For example, a 2016 study focused on trypanothione reductase, a key enzyme in the metabolism of *T. cruzi*. The analysis was performed by molecular docking and binding modes, together with in vivo studies of different alkaloids isolated from plants with wide structural diversity. The results obtained *in silico* were consistent with previously published in vitro results, allowing the structural characteristics of the interaction mode of certain alkaloids with the protein to be defined. The study proposed quebrachamine (**2**), cephalotaxin (**3**), cryptolepine (**4**), (22S, 25S)-tomatidine (**5**), (22R, 25S)-solanidine (**6**), and (22R, 25R)-solasodine (**7**) as new leads for the discovery of potent and selective inhibitors of trypanothione reductase [20].

From the synthetic point of view, the analysis of the rational design of a family of 2-alkylaminomethylquinoline derivatives as antichagasic agents is relevant and representative [21]. In this sense, most of the synthesized compounds showed trypanocidal activity in the different stages of the parasite. Three of them stood out for their SI. The ethyl 2-((4-benzyl-1,4-diazepan-1-yl)methyl)-6-chloro-4-phenylquinoline-3-carboxylate analog (**8**) (IC_50_ = 2.1 mM, SI = 386), in addition to this performance, can prevent tissue inflammation, a key factor in preventing the progression to chronic chagasic cardiomyopathy. Its therapeutic effects, resulting from the optimization of a semisynthetic process based on a natural platform, are very promising.

Finally, a notable example is the repositioning of bioactive compounds through synergistic combinations [22]. The authors corroborated the in vitro synergistic profile by in vivo analysis for the combination of posaconazole (**9**) (traditionally used as an antifungal) and tomatidine (**5**) (a natural steroidal alkaloid with recognized antitrypanosomatid activity by inhibition of C-24 sterol methyltransferase) [23]. These compounds are shown in Figure 1 as “9 + (5)” to refer to the combination of both. The results showed a reduction in parasite load and mortality rates of the animals. The authors attribute this behavior to the simultaneous action on the enzymes responsible for the biosynthesis of sterols, which interferes with the fitness and intracellular metabolism of the parasite. The finding of this synergy encourages further studies on this class of compounds and reinforces the potential for drug repurposing and combination protocols, which represent cost- and time-effective strategies for Chagas disease.

### 2.2. Terpenes

This section highlights the wide chemical diversity of this family of compounds, which includes structures with carbon skeletons of 10 to 30 atoms. Recently, plant terpenoids have been reviewed in the literature for their high value against trypanosomiasis [24].

The isolation of the diterpene 5-epi-icetexan (**10**) from *Salvia gilliesi* has been reported. It is active against epimastigotes at very low concentrations [25]. To delve into its mechanism of action at the molecular level, it was observed that the diterpene 5-epi-icetexone may have multiple effects on the cell cycle of parasites in their intracellular form, slowing down cell division at cytostatic doses. Additionally, it shows in vivo activity in animal models without apparent side effects [26]. The authors suggest that the quinone functional group may be responsible for generating oxidative stress, although further studies are needed to confirm the mechanism.

**Figure 2:**
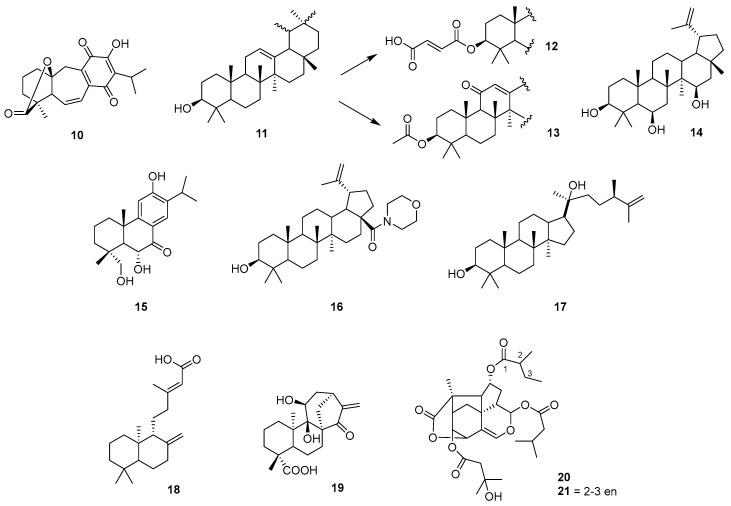
Representative terpenes with anti-*T. cruzi* properties.

One of the best-known and most abundant triterpenes in nature is amyrin. A multidisciplinary study involving the isolation of a binary mixture of α/β-amyrin (**11**) from *Protium* resin reported trypanocidal and cytotoxic activity, along with the mechanisms of action of α/β-amyrin (IC_50_ = 20.2 mM against amastigotes), its semi-synthetic derivatives, and related triterpenoids on trypomastigotes and amastigotes [27]. The authors note that an increase in polarity improves selectivity toward the protozoan. Figure 2 shows the structure–activity relationships (SAR), including derivatives **12**, **13**, and compound **14** (IC_50_ = 3.2, 55.3, 5.9 mM, respectively). This type of compound promotes changes in the mitochondrial membrane potential and ultrastructural features. When combined with benznidazole, it has synergistic effects against amastigotes.

Other representative scaffolds of this family include abietic and betulinic acids. The results obtained for these compounds and their chemical derivatives (**15** and **16**, respectively), both in vitro and in vivo, are consistent with those of other triterpenes and exceed benznidazole in terms of efficacy and low toxicity. These compounds cause morphological alterations and affect the parasites’ energy metabolism [28,29]. Among triterpenoids, dammarane-type compounds isolated from hexane and ethanol extracts of wax “carnauba” (*Copernicia prunifera*) were effective against *T. cruzi* trypomastigotes, with IC_50_ values in the micromolar range (**17**), without showing cytotoxicity at these concentrations [30].

Among diterpenes, oleoresins from different Brazilian *Copaiba* species are good sources. In addition to showing antiparasitic activity, their chemical structures allow modifications that may improve activity (e.g., copalic acid (**18**), IC_50_ = 1.3 mM, against amastigotes). A 2012 study explored the antiparasitic and cytotoxic mechanisms of various diterpenes and established SAR [31]. The authors found that terpenes in *Copaiba* oils can kill parasites through oxidative stress, autophagy, and interference with osmotic regulation. They also concluded that the amastigote forms are more sensitive to terpenes than other life stages. Some compounds showed synergistic behavior, which may occur in nearly all *Copaiba* oils and could explain their medicinal properties.

A recent report on ent-kaurane diterpenoids from the bioactive extract of *Gymnocoronis spilanthoides* var. *spilanthoides* (Asteraceae) reinforces the potential of this class of bioactive compounds as new anti-*T. cruzi* drugs [32]. Adenostemmoic acid B (**19**) was active and selective against the proliferative stages of *T. cruzi* (epimastigotes and amastigotes) and was the most active compound in a murine model of infection, reducing parasitemia and weight loss during the acute phase. Furthermore, treatment with this diterpene in combination with benznidazole increased animal survival. This report also evaluated its immunoregulatory effects, which may enhance host protection by controlling inflammatory reactions associated with the chronic stage of Chagas disease, where current drugs are ineffective. This compound demonstrated strong anti-inflammatory activity in murine macrophages stimulated with lipopolysaccharide and other Toll-like receptor agonists. Similarly, treatment of macrophages with adenosine triphosphate B reduced tumor necrosis factor (TNF) secretion and nitric oxide production, while increasing interleukin-10 (IL-10) levels. The combination of adenostemmoic acid B with benznidazole further inhibited nuclear factor kappa B and reduced nitrite levels. These findings suggest that adenostemmoic acid B is a promising candidate for future studies in the search for new Chagas disease treatments.

More complex diterpenic structures, such as trixikingolides from *Trixis vauthieri* (Asteraceae), exhibit strong in vitro trypanocidal activity (IC_50_ = 0.053 mM for a 4:1 mixture of **20** and **21**) against intracellular trypomastigotes and amastigotes of *T. cruzi.* Although isolated as a mixture, the IC_50_ is about 70 times lower (more potent) than that of benznidazole, positioning it as a candidate for in vivo tests and as a possible prototype for trypanocidal drugs [33].

### 2.3. Sesquiterpenes

A large family of compounds belonging to this type of nucleus is constituted by sesquiterpenes. Because of their remarkable chemistry and bioactivity, they are of great interest in the study of PNOs. A good example is costic acid (**22**), isolated from *Nectandra barbellata* (Lauraceae) through a bioguided study [34]. This compound shows micromolar-order inhibition against trypomastigotes and amastigotes of *T. cruzi*, with high selectivity over intracellular amastigotes. Considering the lethal action of costic acid by affecting a vital and unique organelle, such as the protozoan mitochondria, and given that *in silico* prediction studies such as the Lipinski’s rules of five, pan-assay interference compound, and absorption, distribution, metabolism, excretion, and toxicity (ADMET) properties show no major concerns, it could be considered a candidate for future drug design studies against Chagas disease.

When considering sesquiterpenoid derivatives optimized by semisynthesis, polygodial deserves special mention. The evaluation of polygodial and its natural and synthetic analogues against *T. cruzi* revealed that, although polygodial itself showed limited efficacy, its natural counterpart epipolygodial (**23**) and its synthetically derived Wittig analogues exhibited low micromolar activity against all three forms of the parasite. Furthermore, a synthetic α,β-unsaturated phosphonate compound demonstrated efficacy comparable to clinically approved drugs, such as benznidazole and nifurtimox, and was active against trypomastigotes. These findings highlight the antitrypanosomal potential of polygodial analogs and support further research to identify a lead compound for preclinical development [35].

**Figure 3:**
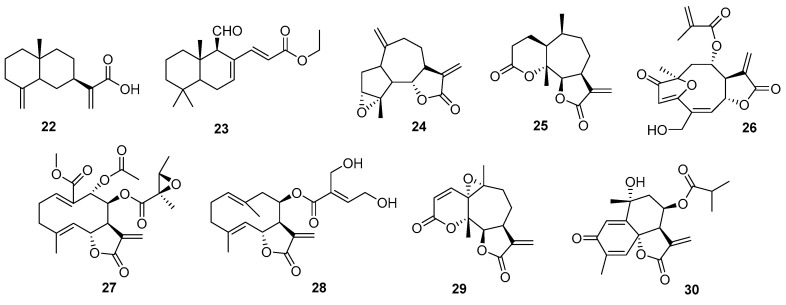
Representative sesquiterpenes and SLs with anti-*T. cruzi* properties.

Without a doubt, within this type of compound, sesquiterpene lactones (SLs) deserve a section. Their marked trypanocidal activity has been reported in numerous articles and reviews [36,37,38,39]. Concentrated in the Asteraceae family, their bioactivity is attributed to the α,β-unsaturated lactone functionality. With numerous skeletons and substitution patterns giving rise to various chemical analogs, they have been evaluated in vitro and in vivo at all stages of the parasite. Although some are markedly cytotoxic at trypanocidal concentrations, many show an SI that ranks them as the leading compounds in the search for antichagasic agents. Figure 3 summarizes some examples of compounds with the most relevant activities (**24**–**28**) [36,40,41,42,43,44].

Our research group recently reported the chemical derivatization of an extract with a high content of SLs, resulting in the isolation of derivatives that outperform the parent compounds and benznidazole in terms of bioactivity and SI, for example, compound (**29**) [37]. Indeed, a similar study on the trypanocidal activity of South American *Vernonieae* extracts and their SLs was recently reported [45]. These two studies report valuable SAR that have not been previously explored for this type of compound. The extracts exhibited good trypanocidal activity, with the greatest effect for pure compounds being exerted by elephantopus-type SLs (from *V. nebularum*) and hirsutinolide-type (from *V. pinguis*), with IC_50_ values of 1.5 and 2.0 mM, respectively. Furthermore, these compounds showed promising SIs (>14).

A recent report refers to the immunomodulatory, antiparasitic, and cardioprotective effects produced by tagitinin C (**30**), alone or in combination with benznidazole, in vitro and in vivo, showing additive effects. When combined, these drugs presented an additive interaction, completely suppressing parasitemia and mediating parasitological cure in all infected mice compared to those treated with benznidazole alone. Furthermore, this combination denotes immunomodulatory and cardioprotective effects by reducing anti-*T. cruzi* immunoglobulin G, pro-inflammatory cytokines interferon gamma (IFN-γ) and TNF-α [46].

### 2.4. Phenolic Acids and Related Compounds

The genus *Stevia* is well known for being one of the “suppliers” of SLs and other bioactive compounds. In this study, the chemical content was analyzed by bioguided fractionation of *Stevia satureiifolia* var. *satureiifolia*. In this case, the isolation of 6-methoxyflavones, such as 5-desmethylsinensetine (**31**) and eupatorine (**32**), previously reported as antiprotozoal nuclei, was achieved [47]. Both epimastigotes and trypomastigotes of *T. cruzi* were shown to be sensitive to these compounds and not toxic to Vero cells, with 5-desmethylsinensetine showing moderate activity against the intracellular amastigote stage [48].

A compound related to salicylic acid (**33**) was isolated from the *n*-hexane extract of *Schinus terebinthifolius* leaves (Anacardiaceae). Together with its hydrogenated derivative, it was shown to be effective in killing the trypomastigote forms of *T. cruzi*, with IC_50_ values < 10 µM and no observed cytotoxicity at the maximum tested concentrations [49]. The promising results led to the investigation of the mechanism by which hydrogenation generated a differential cellular target in parasites. The hydrogenated derivative showed a different lethal effect, affecting the parasite’s mitochondria rather than the plasma membrane, as observed with the parent compound. Therefore, the results suggested that these differences could be associated with the presence of the lipophilic side chain (unsaturated or saturated), since salicylic acid did not show activity against *T. cruzi* trypomastigotes in vitro, leading to the conclusion that bioenergetic systems are of interest for drug discovery against trypanosomatids.

The work published in 2016 by Cornelio et al. describes another strategy for the discovery of trypanocidal compounds [50]. The authors include a possible biological target previously described as part of the bioguided tests, rather than focusing on a particular stage of the parasite. Glyceraldehyde-3-phosphate dehydrogenase is a key enzyme involved in the glycolytic pathway of *T. cruzi*. The bioguided assay on *T. cruzi* glyceraldehyde-3-phosphate dehydrogenase proved to be an efficient method for identifying new inhibitors in complex matrices such as crude plant extracts. From the *Spiranthera odoratissima* species, the flavonoid tiliroside (**34**, kaempferol-3-O-β-D-(6″-trans-p-coumaroyl)-glucopyranoside) was described (reported for the first time in this species) as an excellent enzyme inhibitor.

### 2.5. Coumarins

**Figure 4:**
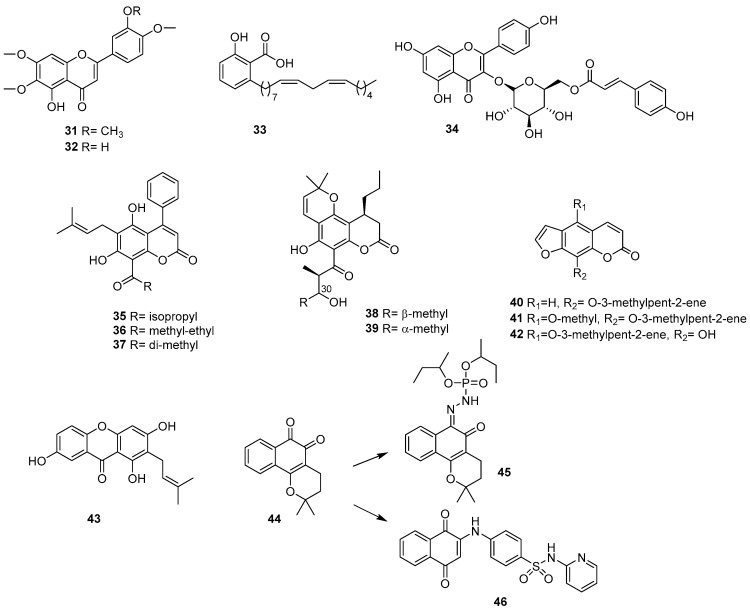
Representative phenolic acids, related compounds, coumarins, and chemical derivatives with anti-*T. cruzi* properties.

Among the phenolic or polyphenol compounds, coumarins are among the most abundant PNOs (Figure 4). They are interesting metabolites from several perspectives, due to their great chemical diversity, variety of reported biological activity, occurrence from natural sources, and ease of synthetic modification and derivatization [51].

Among these types of compounds, those with a 2H-1-benzopyran-2-one skeleton, such as mammea-type isolated from the leaves of the tropical tree *Calophyllum brasiliense* (Calophyllaceae), have shown activity against a Guatemalan strain of *T. cruzi* TcI H6 [52]. Mammea A/BA (**35**) and the coumarin mixture (**36**, **37** in different proportions) showed high trypanocidal activity and a high SI (up to four times more active than benznidazole in three Mexican strains of *T. cruzi*) [53]. These compounds induced serious physiological and morphological alterations, affecting the mobility, growth recovery, and ultrastructure of epimastigotes (with severe alterations in the plasma membrane and nuclear envelope), and drastically reducing the infectivity of trypomastigotes in Vero cells. The results presented in this report suggest these compounds as potential candidates for preclinical studies.

From the stem bark of the same species, a bioguided fractionation of the methanolic extract was performed, leading to the isolation of two related coumarins: calanolides E1 (**38**) and E2 (**39**), which were active against *T. cruzi* with half-maximal effective concentration (EC_50_) values in the micromolar range [54]. These compounds are isomeric and show different SIs, leading the authors to suggest that the C-30 configuration plays a determining role in parasite inhibition.

After the initial extensive trypanocidal screening of Apiaceae plant species (see the section in which the activity of the extract is analyzed), bioactivity-guided fractionation and bioassay-guided isolation were carried out on four selected antitrypanocidal crude ethyl acetate extracts. Antichagasic dihydropyranochromens and pyranocoumarins were identified, and the synergistic effects of the mixtures, selectivity, cytotoxicity, and implications for the biological pathways of host cells involved in parasite replication and immune evasion were analyzed. Therefore, Krishnan et al. reported that a defined mixture of furanocoumarins (imperatorin (**40**), phellopterin (**41**), and alloimperatorin (**42**)) acted synergistically to significantly inhibit parasite replication and/or release (approximately 80% inhibition at 10 μg/mL) in infected mammalian cells. Interestingly, when individually tested at 10 μM, pure furanocoumarins did not show comparable trypanocidal activity, suggesting a possible case of synergistic interaction between these natural compounds, although this phenomenon was not further explored [55].

### 2.6. Quinones

This family of compounds is widely distributed in nature, mainly in plants, although they are also found in fungi, bacteria, and even animals. Within this family, naphthoquinones are particularly important due to the diversity of biological activities they have demonstrated, such as antimalarial, anticancer, and antifungal properties, among others [56]. This work evaluated the activity, ultrastructural, and morphological alterations induced by xanthone 1,3,7-trihydroxy-2-(3-methylbut-2-enyl)-xanthone (**43**), isolated from *Kielmeyera coriacea*, against *T. cruzi* [57]. It inhibits the three forms of the parasite, being more active against intracellular amastigotes, without inducing toxicity in mammalian cells, surpassing benznidazole and most of the xanthones previously published in the literature. The available data suggest that the trypanocidal action of this xanthone may involve an imbalance in the redox metabolism of the parasite, altering the mitochondria (a specific target of trypanosomatids) by increasing the production of O_2_^•−^.

β-lapachone (**44**) is one of the most important antiparasitic naphthoquinones isolated from different plant families [58]. Furthermore, its analogues exert multifactorial effects on various targets of antiparasitic chemotherapy and show improved activity on *T. cruzi*. A representative work in this regard is that published by Oliveira dos Anjos et al., where the effects of a new β-lapachone derivative (**45**) on the survival and proliferation of *T. cruzi* were evaluated, along with the mechanisms underlying the death of the parasite [59]. Its SI indicates that the compound is ten times more cytotoxic to epimastigotes than to macrophages or splenocytes. The analyses carried out demonstrate that the β-lapachone derivative affects the morphology and topography of the surface of *T. cruzi*, selectively triggering the cell death of the parasite, which involves both apoptosis and autophagy-induced necrosis.

Another interesting aspect of these types of compounds is their versatility in bioinformatic analyses on different target proteins. In this sense, cruzain is one of the main cysteine proteases in *T. cruzi*, performing essential functions for the parasite’s survival. It is involved in the process of invasion, differentiation, and proliferation in host cells, thus providing an attractive target for the development of a broad-spectrum inhibitor for the treatment of Chagas disease. In this context, 26 cruzain 3D structures were evaluated by molecular redocking to identify the most appropriate target. Subsequently, a virtual screening of a library of about 120 natural and non-natural molecules was carried out. Fourteen naphthoquinone-like analogs were identified, synthesized, and evaluated. Of these, three compounds were active against cruzain, with a 1,4-naphthoquinonepyridin-2-ylsulfonamide derivative (**46**) being the most active molecule (IC_50_ = 6.3 µM) [60]. The results of docking, energy decomposition (molecular mechanics, Poisson–Boltzmann surface area), and molecular dynamics suggest a stable complex with van der Waals interactions as the most relevant.

**Figure 5:**
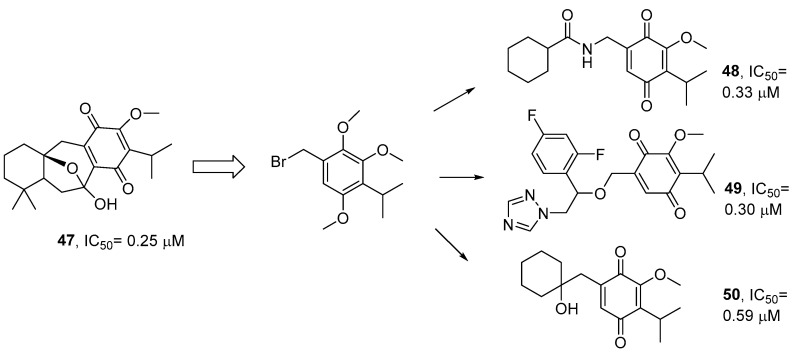
Komaroviquinone structure and chemical synthetic precursor for quinone trypanocidal derivatives.

Following this line of work, a representative publication on the rational design and synthesis of quinone derivatives evaluated synthetic intermediates of komaroviquinone (**47**) as trypanocides, with the aim of obtaining SAR, which is useful when synthesizing more effective and less toxic-related compounds [61]. Based on the reported trypanocidal activity for komaroviquinone [62], isolated from *Dracocephalum komarovii*, the rational design of its total synthesis allowed the production of synthetic intermediaries and valuable simplified quinone-type derivatives, which are useful when establishing SAR. Many of them showed greater antiprotozoal activity against *T. cruzi* trypomastigotes than benznidazole, without concomitant toxicity to the host cell. As a summary, the most active synthesized analogues (**48**, **49**, **50**) are shown in Figure 5. Through this exhaustive multidisciplinary work, the authors conclude that the quinone moiety of komaroviquinone plays an important role in its antitrypanosomal activity, but the complex and fused cyclic structure is not necessarily always essential. Although previous studies postulate that the quinone residue of komaroviquinone participates in the generation of reactive oxygen species within the pathogen [63], in this case, it is necessary to explore the efficacy of these compounds in vivo.

### 2.7. Lignans

**Figure 6:**
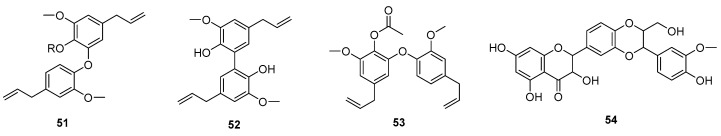
Lignan and chemical derivatives: representative structures.

In this section, compounds such as dehydrodieugenol and silibinin are presented as representative examples (Figure 6). Dehydrodieugenol (**51**) was isolated from the hexanic extract of *Nectandra leucantha* (Lauraceae) leaves. In 2015, an improvement in antiparasitic potential was reported following the introduction of an additional methyl group in related structures (**52**) [64]. Based on these findings, a methylated derivative of this neolignan (dehydrodieugenol dimethyl ether, **53**) was synthesized. Both compounds showed potent antitrypanosomal activity against intracellular trypomastigotes and amastigotes [65]. Dehydrodieugenol induced rapid plasma alteration in *T. cruzi*, leading to parasite death. Both compounds eliminated parasites within macrophages through mechanisms independent of reactive oxygen and nitrogen species.

These types of compounds have been the subject of synthetic modification, leading to the generation of a family of chemical derivatives aimed at exploring the SAR of synthetically accessible compounds against *T. cruzi*. Among these, a phenolic acetate derivative of dehydrodieugenol B emerged as the most effective compound in the series against both forms of the parasite [66]. This compound induced a rapid and intense depolarization of mitochondrial membrane potential, accompanied by decreased levels of intracellular reactive oxygen species. SAR studies demonstrated that the presence of at least one allyl side chain in the biaryl ether core was important for antitrypanosome activity, and that a free phenol is not essential.

Silibinin (**54**) is a natural compound isolated from the silymarin complex of isoflavolignans extracted from the seeds of *Silybum marianum* (Asteraceae), with reported antifungal, anti-inflammatory, and antiprotozoal activity. In addition, it inhibits the efflux pump (Pgp) in host cell membranes and induces death in trypanosomatids. Recently, its remarkable activity against *T. cruzi*, along with low in vitro and in vivo cytotoxicity when used in combination with benznidazole, has been reported. Monotherapy with silibinin failed to control parasitemia and mortality in animals, reinforcing the concept of using synergistic combinations with this type of compound [67].

### 2.8. Marine PNOs

The bioprospecting of macro and marine microorganisms has led to the discovery of novel and complex compounds with therapeutic potential. Due to the complexity and hostility of the environment in which marine species develop, these organisms have evolved a wide range of bioactivities to ensure their survival and serve as chemical defenses [68,69].

**Figure 7:**
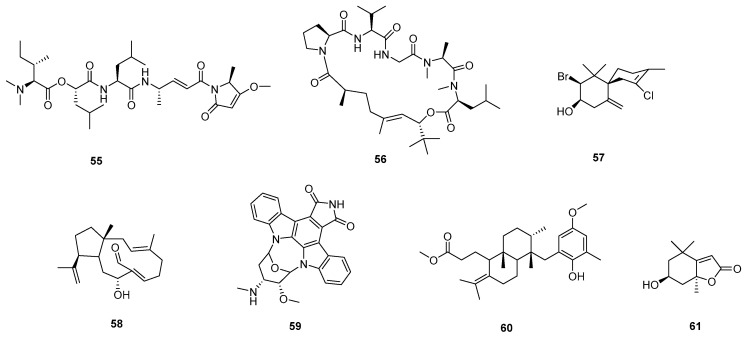
Summary of the most representative marine compounds isolated in the decade as trypanocides.

The chemical diversity by the study of natural marine products has enabled the isolation of novel antiparasitic compounds, such as alkaloids, terpenoids, peptides, polyketides, terpenes, coumarins, steroids, fatty acid derivatives, and lactones [70]. For these compounds, general observations include the absence of cytotoxicity in host cells, submicromolar potencies, and a lower possibility of inducing drug resistance. However, the authors emphasize a lack of systematic studies in this area, along with a pressing need for pharmacokinetic and pharmacodynamic evaluations. Figure 7 summarizes and illustrates the structural diversity of the most active marine compounds reported in the past decade (**55**–**61**).

### 2.9. Extracts and Herbal Drugs

This section discusses the use of complete extracts, bioactive fractions, or pure compounds isolated from activity-guided fractionation studies of trypanocidal extracts. Among the numerous reports reviewed, some have performed bioguided fractionation not only of the “parent” extract but also on fractions of varying polarity, which allows a broad segregation of the compounds (not less valid for that reason). On many occasions, a masking of the biological activity occurs due to the concentration of certain compounds in the mixture or due to antagonistic interactions. It is also necessary to highlight the importance of a large-scale screening, including preliminary studies of extracts with potential trypanocidal activity, which have historically led to the discovery of new therapeutic agents. In this sense, many authors point out that polar compounds, such as glycosides, polymers, or polyphenols, potentially present in botanical drugs, often exhibit poor systemic bioavailability. Likewise, the in vitro profile of compounds isolated through bioguided fractionation can yield false negatives, as secondary metabolites are metabolized in vivo and may act as inactive prodrugs.

In addition, it is necessary to consider and evaluate the possible additive, synergistic, or antagonistic effects of the PNOs present in plant extracts.

South America contains many ecosystems with native species and the highest levels of biodiversity in the world. In this context, a cross-sectional ethnopharmacological field study was conducted in Bolivia, where Chagas disease is hyperendemic, to identify phylobioactive hotspots. In this multidisciplinary study, a total of 775 botanical drug extracts (used in traditional medicine either for Chagas disease or for unrelated indications) were profiled, leading to the discovery of different classes of natural anti-chagasic products [71].

The species *Senna chloroclada*, used in ethnobotany as an antichagasic therapy, proved to be the source of a phylobioactive hotspot (anthraquinones). *S. chloroclada* flower extract showed significant inhibitory effects on parasite release in the infection assay (15 mg/mL was as effective as 20 mM benznidazole) without cytotoxicity to host cells. Other highly active and selective antitrypanosomal extracts included those from *Pterodon* sp. seeds, leaves of *Sonchus oleraceus*, aerial parts of *Acanthostyles buniifolius*, *Aloysia polystachya*, and *Gloxinia gymnostoma*.

**Figure 8:**
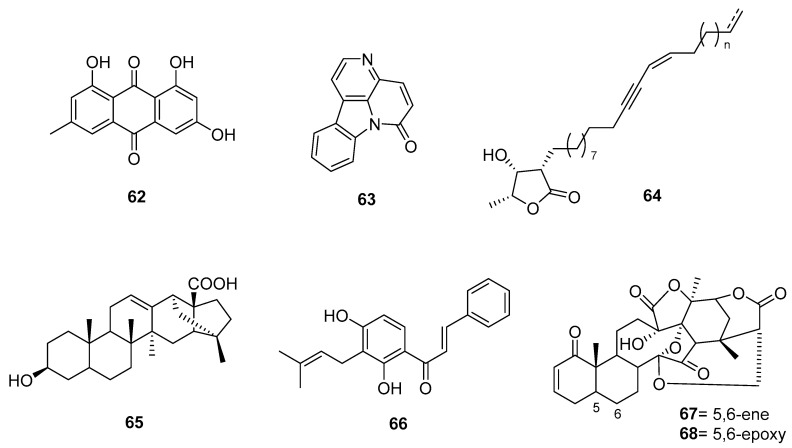
Representative compounds isolated from bioguided fractionation from trypanocidal vegetal extracts.

Common 9,10-anthracenedione derivatives were also tested in *T. cruzi* cell infection assays, identifying hydroxyanthraquinone as a possible antichagasic scaffold (**62**) (Figure 8). This type of study leads to the systematic identification of antichagasic agents in the plant kingdom and lays the groundwork for future phytochemical research and bioprospecting.

In a screening of methanol and dichloromethane extracts from Argentine species of the Asteraceae family against *T. cruzi* epimastigotes, the most noteworthy results at 10 µg/mL were observed for extracts of *Aspilia silphioides* and *Thelesperma megapotamicum* (dichloromethane), with growth inhibition percentages of 93.4 and 95.6%, respectively [72].

Ferreira and colleagues proposed the first “sustainable treatment for Chagas” using ethanolic leaf extracts of *Zanthoxylum chiloperone* (Rutaceae), which showed comparable in vitro activity against both trypomastigote and amastigote forms of *T. cruzi* as benznidazole. The main isolated alkaloidal, canthin-6-one (**63**), was also active [73,74].

Regarding the vast Brazilian flora, a study on eight species from the Cerrado biome evaluated 37 extracts of varying polarity, 24 of which showed antiparasitic effects. For activity against *T. cruzi* amastigotes, the hexanic root extract of *Vatairea macrocarpa* (Fabaceae) had an IC_50_ = 36.2 μg/mL with no apparent cytotoxicity toward L6 cells (IC_50_ > 100 μg/mL) [75].

In line with exploring Brazilian flora for new anti-*T. cruzi* compounds, the methanolic (MeOH) extract from the fruit peels of *Porcelia macrocarpa* (Annonaceae) exhibited strong activity against both trypomastigotes and intracellular amastigotes and was subsequently subjected to bioactivity-guided fractionation [76]. Through multiple chromatographic steps, a fraction containing a mixture of four newly identified, structurally related acetogenin-type compounds (**64**) was isolated. This mixture showed EC_50_ values of 4.9 µg/mL and 2.5 µg/mL against trypomastigotes and amastigotes, respectively, comparable to benznidazole (EC_50_ of 4.8 µg/mL and 1.4 µg/mL). Furthermore, it did not exhibit toxicity toward murine fibroblasts, yielding a high SI. The mechanism of action was investigated: while short-term exposure did not affect cell membrane permeability, it reduced intracellular calcium levels without altering the acidocalcisome pH. These effects were accompanied by increased reactive oxygen species and ATP levels, without mitochondrial membrane depolarization. Collectively, these results suggest that the acetogenin mixture induces irreversible oxidative stress, ultimately leading to parasite death. If further studied, these compounds could offer valuable insights into novel cell death pathways in *T. cruzi*, opening avenues for new therapeutic strategies.

Brazilian nutraceutical species of the Myrtaceae family, such as *Psidium brownianum*, showed excellent activity against *T. cruzi*, but were toxic to fibroblasts [77]. *Eugenia uniflora* (pitanga) was reported as a promising alternative due to its low in vitro toxicity [78].

The genus *Pfaffia* (Amaranthaceae), comprising around 22 species in Brazil and marketed as Brazilian ginseng, was also investigated. A study reported anti-*T. cruzi* from the hydroalcoholic root extract of *Pfaffia glomerata*, its hydrolyzed fractions, and pfaffic acid (**65**). One hydrolized fraction presented an IC_50_ of 47.89 μg/mL and contained campesterol, stigmasterol, β-sitosterol, Δ^7^-stigmastenol and Δ^7^-spinasterol, fatty acid esters, and aliphatic hydrocarbons as major components [79].

Another Brazilian species with notable antiparasitic efficacy is *Lonchocarpus cultratus* (Fabaceae). In a 2021 study using complete bioguided fractionation, dichloromethane and hexane extracts significantly reduced parasite numbers (at 175 mg/mL, growth inhibition was 99.32% and 95.96%, respectively). The dichloromethane extract showed higher activity across all tested *T. cruzi* (epimastigotes, trypomastigotes, and amastigotes) with IC_50_ values close to benznidazole (10.98, 2.42, and 0.83 mg/mL, respectively). This activity was attributed to chalcones (such as derricin, lonchocarpine, isochordoine (**66**)) and terpenes. The extract showed acceptable selectivity in macrophages, making this species a promising candidate for future explorations [80].

Piperaceae species are abundant in the tropics and play key ecological roles. Extracts from this family have shown wide-ranging bioactivity. Regarding essential oils of Piper, for species such as *P. tuberculatum* [77], *P. diospyrifolium*, and *P. mikanianum* [81], studies have reported antiparasitic potential linked to phenylpropanoid-rich compositions. Polar leaf extracts from *P. marginatum*, *P. subpedale*, and *P. jericoense* also showed high activity against *T. cruzi* with low toxicity (not cytotoxic, mutagenic, or genotoxic).

In a broader context, a 2015 study analyzed 58 extracts of 53 approved and marketed herbal medicines in Germany using multi-target screening against *Leishmania*, *Trypanosoma*, and *Plasmodium* [82]. Sixteen extracts showed in vitro activity (IC_50_ < 10 µg/mL). The extracts were analyzed using ultra-high-performance liquid chromatography with electrospray ionization quadrupole time-of-flight mass spectrometry and tandem mass spectrometry. For *T. cruzi* amastigotes, ethanolic extract of *Valeriana officinalis* L. (Caprifoliaceae) was identified for the first time as active (IC_50_ = 5.86 µg/mL; SI = 7.9). Given its widespread cultivation, valerian represents a promising new source of therapeutic agents against *T. cruzi.*

A study on ethanolic extracts of representative Colombian flora identified flavonoids and triterpenoids in *Clethra fimbriata* (Cletharaceae) as responsible for 50.5% growth inhibition of *T. cruzi* trypomastigotes at 100 μg/mL. Extracts from *Ageratina vacciniaefolia* and *Siparuna sessiliflora* also showed activity against epimastigotes with low mammalian cytotoxicity. *C. fimbriata* additionally stimulated IFN-γ and TNF-α secretion by CD8^+^ T cells, indicating its potential to combine trypanocidal activity with immunomodulatory effects [83].

In a similar screening of methanolic extracts from ten Mexican species traditionally used as antiparasitics (Artemisia mexicana, Castela texana, Cymbopogon citratus, Eryngium heterophyllum, Haematoxylum brasiletto, Lippia graveolens, Marrubium vulgare, Persea americana, Ruta chalepensis, and Schinus molle), *E. heterophyllum*, *H. brasiletto*, *M. vulgare*, and *S. molle* showed the strongest trypanocidal activity (88–100% inhibition at 150 mg/mL). While phytochemical studies exist for some, further work is needed to confirm whether their main isolated components are responsible for activity [84].

The chloroformic bark extract of the tropical species *Helietta apiculata* [85] showed moderate in vivo efficacy in mice infected with *T. cruzi* (42 to 54% parasitemia reduction over 60 days) without side effects or mortality.

A notable case is bee propolis [86], which has been increasingly studied for its antiparasitic effects [87]. A deeper chemical analysis of propolis may reveal structures similar to many described in this review. Comprehensive in vitro and in vivo studies suggest that its activity relates to aromatic acids and flavonoids, which inhibit *T. cruzi* proliferation [88]. Importantly, these effects are primarily observed in crude extracts tested, not purified compounds.

The Amaryllidaceae family has a long history of biologically active compounds. An extensive screening of 79 extracts against *T. cruzi* identified two highly active species: *Crinum erubescens* and *Rhodophiala andicola.* Both showed low cellular toxicity in Vero cells, with SI > 37 [89].

Finally, an exemplary study conducted in 2015 on *Physalis angulate* [90], historically used as an antiparasitic, highlights its bioactive steroids physalins B and F (**67** and **68**, respectively), which show strong anti-*T. cruzi* activity [91]. Due to low yields and high isolation costs, the ethanolic extract of *P. angulate* is proposed as an alternative. It is stable, low in toxicity, nonmutagenic, and readily accessible. The extract effectively inhibited epimastigote growth and was highly toxic to trypomastigotes and amastigotes. Mechanistically, it reduced parasite load by disrupting the cell cycle and inducing necrotic cell death. Furthermore, it was able to reduce the course of an acute infection in vivo (murine model). Notably, the extract displayed synergy with benznidazole in vitro, showing a promising therapeutic profile.

Returning to the goal of highlighting the therapeutic potential of PNO, the study of raw and fractionated plant extracts is both invaluable and essential. Therefore, collaborative efforts are crucial to exploring biodiversity in the search for new anti-*T. cruzi* compounds.

## 3. Antimalarials PNOs

### 3.1. Alkaloids

**Figure 9:**
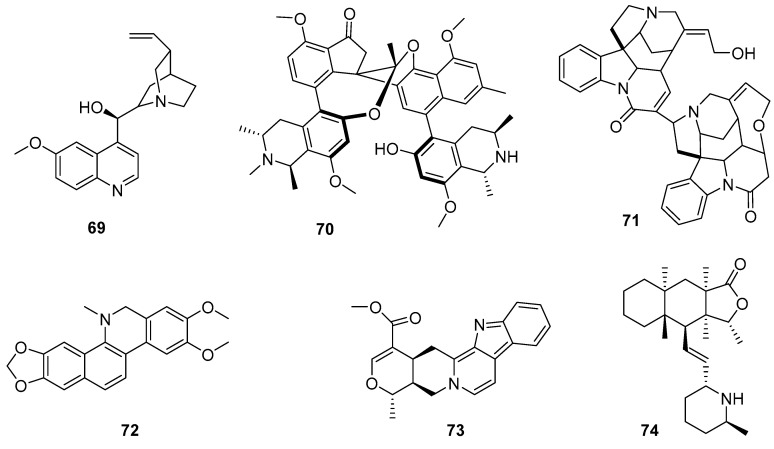
Antimalarial representative alkaloids.

Without a doubt, quinine (**69**) (Figure 9) is the representative alkaloid in the fight against malaria. Since the seventeenth century, it has been isolated from the bark of *Cinchona* and used to treat malaria since the 1600s. It has rapid schizonticidal action against the intraerythrocytic parasites of malaria; it also has analgesic properties, but not antipyretic. Its antimalarial mechanism of action is unknown, and while it remains an important antimalarial drug nearly 400 years after Jesuit priests first documented its efficacy, its use is restricted to mild and simple cases of this disease. This is mainly due to its toxicity, poor compliance with treatment by patients, and the implementation of newer and better-tolerated therapies such as artemisinin (ART)-based combination therapy [92].

Countless reports and reviews show that plant-derived alkaloids are among the most promising compounds in terms of biological activity [93]. Some representative examples of the complexity and structural diversity of these compounds are spirombandakamine A3 (**70**) (a dimer of spiro-fused naphthylisoquinoline) isolated from a Congolese plant of the *Ancistrocladus* family (IC_50_ = 0.071 mM against *P. falciparum* and SI = 272) [94]. This work represents a great effort in terms of structural elucidation and provides chemotaxonomic clues to the classification of previously undescribed plant species. Another example is strychnogucine B (**71**), a bisindole alkaloid isolated from *Strychnos icaja*, which also has very promising in vitro antiplasmodial properties. Furthermore, this compound was shown to be synthetically accessible from (−)-strychnine in four steps [95]. Also exhibiting outstanding antimalarial activity is the steroidal alkaloid conessine, which is isolated from the bark of *Holarrhena antidysenterica* [96], and bisbenzyl isoquinolinics, such as cycleanine and its derivatives, which are isolated from *Triclisia subcordata* (with activity in the nanomolar range against chloroquine-sensitive and resistant *P. falciparum*) [97]. Benzophenatridine-type alkaloids, such as dihydronitidine, pelitorin, and heitziquinone, were isolated from the bark of *Zanthoxylum heitzii.* In this sense, the outstanding activity and efficacy of dihydronitidine (**72**) (in the sub-nanomolar range) is counterbalanced by its reduced extractability (which limits its concentrations in aqueous preparations of *Z. heitzii*), making it a good candidate for use in preparations with nonpolar extraction methods or simply by using powdered bark [98].

A recent study highlights the urgent need to discover new chemical agents with unique mechanisms of action for both the treatment and prevention of malaria. While therapeutic drugs must act quickly to remove parasites and alleviate clinical symptoms, slower-acting compounds can serve as viable options for malaria prophylaxis. In this study, an in vitro screening against *P. falciparum* of 424 pure compounds derived from PNOs was conducted. Only 46 compounds show > 50% inhibition at 10 µM. Dose–response assays revealed nine compounds with submicromolar effects and high SIs, with confirmed slow-acting activity for two alkaloids: alstonin (**73**) and hembelin (**74**). Cross-resistance studies with different lines of *P. falciparum* suggest that these alkaloids act differently than currently used antimalarials, which requires more research on the molecular targets and modes of action of these compounds [99].

### 3.2. Terpenoids

**Figure 10:**
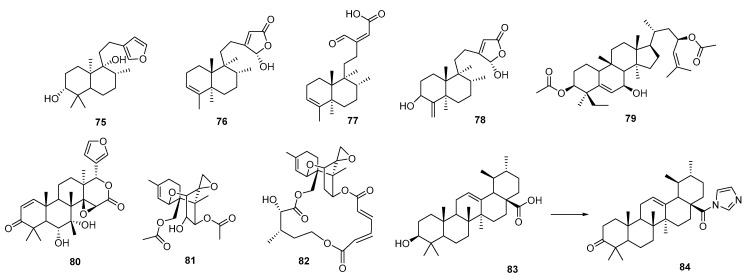
Most antimalarial representative natural terpenoids and chemical derivatives.

Within this vast family of compounds, diterpenes such as the labdane-type compound otostegindiol (**75**), isolated by bioguided fractionation of the *Otostegia integrifolia* species (a plant widely used in traditional Ethiopian medicine against malaria), showed potent antiplasmodial activity against *P. berghei*, without significant toxicity, and demonstrated relative safety when administered orally [100]. Another species used in folk medicine in Ghana as an antimalarial is *Polyalthia longifolia* var. *pendula*. Bioguided fractionation of its ethanolic extract led to the isolation of three clerodan-type diterpenes (**76**, **77**, **78**, shown in Figure 10), a steroid, and two alkaloids. These clerodane diterpenes exhibited significantly potent schizontocidal activity in the blood (IC_50_: 3–6 mg/mL). Interestingly, these diterpenes antagonize the antiplasmodial activity of chloroquine, suggesting a possible herb–drug interaction that may result from the concomitant administration of *P. longifolia* phytomedicines and conventional drugs such as chloroquine [101]. These results highlight the importance of analyzing the multiple variables involved in the study of phytomedicines.

Numerous reports have documented the significant antimalarial activity of triterpenes. An interdisciplinary study in which 21 triterpenoids (either isolated from aerial parts of the African medicinal plant *Momordica balsamina* or obtained by chemical derivatization) were evaluated for their activity against the liver stage of *P. berghei* [102]. One of the derivatives (**79**) exhibited significant activity at the lowest concentration used (1 µM), without toxicity. This compound is a strong inhibitor of *P. falciparum* and shows dual-stage antimalarial activity.

An interesting group of metabolites within this family is the limonoids. In 2014, Pereira et al. [103] isolated and chemically derivatized a series of these compounds from the *Carapa guianensis* species. This cultivable tree is traditionally used by practitioners in the Amazon region to treat malaria-related symptoms. The importance of the work lies not only in its results but also in the fact that the compounds were isolated from the abundant residual pressed seed material (a waste by-product of carapa or andiroba oil production). Limonoids (**80**) and their derivatives exhibited moderate micromolar inhibition concentrations, with good SI, and allowed the identification of key structural features associated with their antimalarial activity.

Among this diverse group of compounds, trichothecenes are tricyclic sesquiterpenoid mycotoxins characterized by their unique structural complexity and notable biological activity. A recent study focused on two trichothecenes: anguidine (**81**) (a non-macrocyclic compound isolated from *Fusarium* species with known total synthesis) and verrucarin A (**82**) (a macrocyclic derivative), due to their potential to target both the blood and liver stages of *Plasmodium* species [104]. Both compounds demonstrated strong antiplasmodial activity; however, they also exhibited significant cytotoxicity, indicating a lack of selectivity that limits their viability for therapeutic development. Nonetheless, their direct interference with the parasite’s protein synthesis machinery suggests their potential utility as chemical probes for studying protein synthesis inhibition.

Finally, some works have reported the isolation of a triterpene known as ursolic acid (**83**, UA) from plant species, followed by a chemical derivatization to improve its antimalarial properties. UA was isolated from the leaves of *Mimusops caffra* (Sapotaceae), a species used by traditional Zulu healers, through a bioguided study [105], and also from *Malus domestica* (apple peel), for synthetic purposes [106]. Figure 10 summarizes the structures of the most active UA derivative (**84**). The results of these works validate the use of these plants in traditional medicine.

### 3.3. Artemisinin

ART (**85**, an SLs) undoubtedly deserves a special section. It was first extracted from the Chinese medicinal plant *Artemisia annua* L. in 1972 by Youyou Tu, who was awarded the Nobel Prize in 2015 for this discovery, which has saved the lives of millions of people around the world [107].

With a complex tetracyclic nucleus that includes an endoperoxide bridge, its chemical structure was elucidated in 1979. ART and its derivatives are currently the first-line treatment for malaria. They are active against multiple strains of the parasite due to their structural uniqueness and act faster than other available drugs. However, they have low bioavailability and a short half-life, which is why combination therapy is preferred over monotherapy. This approach reduces treatment time and lowers the probability of resistance development. Because of this, it is one of the most studied compounds of natural origin in human history.

In 2015, a group of researchers demonstrated that oral administration of the whole plant of *A. annua* (not infusion or tea) [108,109] led to slower development of resistance in parasites compared to the pure metabolite, in mice infected with *P. yoelii*. This increased the effective therapeutic lifespan of the treatment. Synergistic benefits may arise from the presence of other antimalarial compounds. The authors attribute the resilience shown by the whole plant to the evolutionary refinement of the plant’s secondary metabolites into a redundant multicomponent defense system. This important finding highlights the need to further explore the role of nonpharmaceutical forms of *A. annua* in malaria treatment. If proven clinically effective and well tolerated, this approach could offer an economical and effective solution for global public health efforts aimed at reducing malaria-related morbidity and mortality, especially since the plant can be grown and processed locally.

In the context of key therapeutic issues, a recent review aims to guide the strategic exploration of antiplasmodial PNOs from *Artemisia* species [110]. The authors discuss the role of ART in the bioactivity of *Artemisia* infusions against specific stages of the parasite (alone or in combination with other phytochemicals). They also focus on the intricate mechanisms of action and biological targets in *Plasmodium* affected by the numerous compounds found in *Artemisia* infusion, with an emphasis on drug-refractory stages of *Plasmodium*, namely hypnozoites (asexual blood stage and liver stage) and gametocytes (sexual stage).

**Figure 11:**
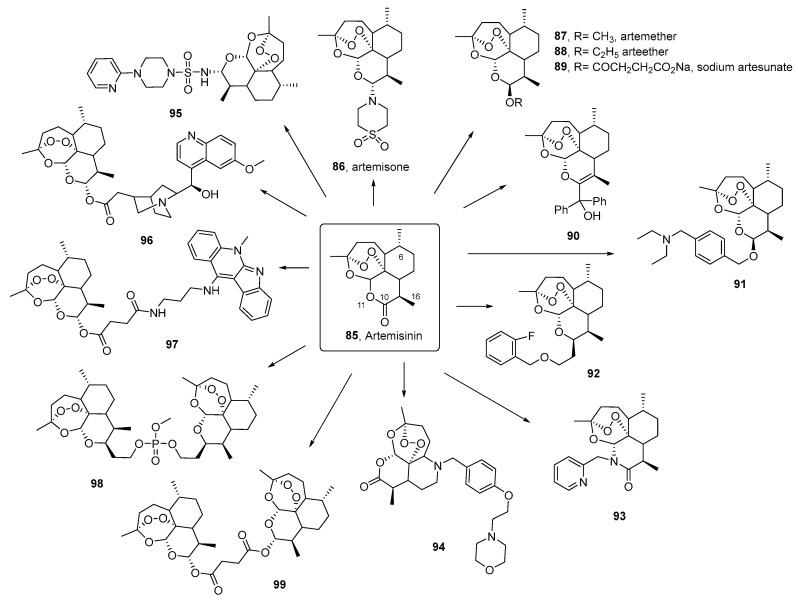
Artemisinin and chemical derivatives with excellent antimalarial properties.

Regarding the synthesis of ART derivatives, an excellent and in-depth review was recently published, compiling the antimalarial activity of both ART derivatives and synthetic peroxides [111]. To date, derivatives with modifications at positions 10, 11, 16, and 6 have been synthesized, along with hybrids/dimers/trimers/tetramers of ART, 1,2,4-trioxolane (ozonides), 1,2,4-trioxanes, and 1,2,4,5-tetraxanes. These compounds exhibit key structural features and have been analyzed for important SAR. Many show potent activity (in the nanomolar range) against various drug-resistant and sensitive *Plasmodium* strains, with excellent SI. See summary Figure 11, inspired by this review, which presents some of the most bioactive and representative derivatives based on their structural motifs.

### 3.4. Quinone, Polyphenolic, and Chromane-Related Compounds

**Figure 12:**
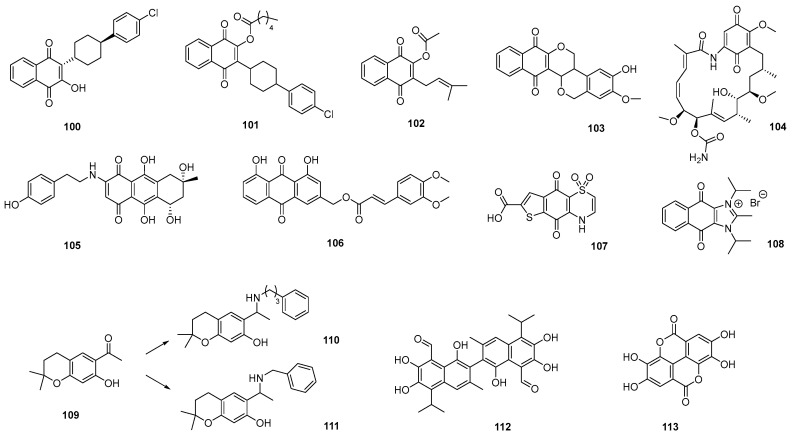
Antimalarial quinone, polyphenolic, and chromane chemical derivatives.

Numerous quinone-derived compounds have attracted considerable attention in recent decades due to their potential for the discovery of antimalarial drugs. Atovaquone (**100**) belongs to a class of naphthoquinone-based drugs used in combination with proguanil (Malarone™) for the treatment of acute, uncomplicated malaria. Over the years, various semisynthetic derivatives of natural quinones have been explored and fully synthesized (naphthquinones, benzoquinone, anthraquinones, thiazinoquinones), and, as in the case of ART, hybrid quinone-based compounds have also been constructed. An excellent current, thorough review documents these efforts. Figure 12 summarizes several quinone-based compounds for their excellent activity and SI (in the micro- and nanomolar range). Most of the selected compounds are chemical derivatives of quinones of natural origin (**101**–**108**). Despite these excellent results, they have not yet been explored in preclinical studies against malaria parasites. This may be due to general challenges that must be addressed, such as toxicity, physicochemical properties, and bioavailability, which are essential for the development of effective preclinical candidates [112].

Furthermore, chromanes and chromenes represent a widely distributed class of PNO. They are heterobicyclic compounds formed by the fusion of an aromatic ring with a dihydropyranic ring (chroman) or a pyranic ring (chromenes). Many of them exhibit high antiprotozoal activity. In 2013, Harel et al. [113], starting from a natural, unstable, and inactive chromene (encecalol angelate) isolated from the extract of *Ageratum conyzoides*, described a synthetic intermediate (encecalin, **109**), from which semisynthetic analogs with promising activity were obtained (**110**–**111**). The most active against *P. falciparum*, with IC_50_ values in the range of chloroquine, exhibited high SI. This study enabled the description of a new class of very potent antiprotozoal compounds.

A recent study has renewed interest in gossypol (**112**), a natural compound derived from cottonseed, previously recognized for its strong antimalarial activity against chloroquine-resistant and -sensitive *Plasmodium falciparum* strains [114]. Although historically promising, gossypol was abandoned due to concerns over toxicity, now understood to be closely related to dosage and duration of treatment. This study evaluated the antimalarial efficacy of gossypol by testing it against clinical and laboratory isolates of *P. falciparum*, providing strong supporting evidence. Notably, the absence of cross-resistance with existing antimalarials suggests that gossypol may target a distinct biological pathway, positioning it as a promising candidate for further drug development. Future research should involve genome-wide and epigenetic studies to better understand its mechanism of action and potential resistance pathways, as well as to uncover novel targets for antimalarial drugs.

A recent study investigated the antimalarial potential of extracts and compounds from over 100 plant species used in traditional Korean medicine. *Geranium thunbergii* was identified for its high antimalarial activity. Among the bioactive compounds, ellagic acid (**113**) displayed the most potent antimalarial activity, with an IC_50_ of 1.60 ± 0.09 µM (on *P. falciparum* strain 3D7) [115]. Previous reports demonstrated significantly higher activity (in the nanomolar range) for this compound, with increased susceptibility in *P. falciparum* strains resistant to chloroquine and mefloquine (F32, Dd2, FcB1, W2, and FcM29). Furthermore, this polyphenol has shown potential as a therapeutic agent for hemoparasites in general and for reducing oxidative stress. Therefore, the development of ellagic acid derivatives is necessary to improve safety and antihemoparasitic activity.

### 3.5. Marine PNO

The extensive chemical diversity of compounds found in the marine environment has attracted considerable attention for drug development. In turn, it is undeniable that the scarcity of these natural metabolites has stimulated efforts toward their total synthesis. Excellent current reviews highlight that the area of marine PNOs with antimalarial potential contains many representative examples of these developments [69,116].

We will expand on two specific reports. In 2011, Conroy et al. reported the total synthesis of gallinamide A (**55**), which, after its structural characterization, was found to be identical to the secondary metabolite symplostatin 4, isolated and identified independently from *Symploca* sp. [117]. This natural marine product has been recognized as a potent antimalarial agent (and antiprotozoal, as previously noted), showing a reduction in parasitemia at nanomolar concentrations. Its mode of action is not yet clear, although it has been shown to inhibit *P. falciparum* falcipains in infected red blood cells, suggesting a role in the inhibition of hemoglobin degradation [118].

A recent report described the isolation and structural determination of palstimolide A (**114**), a complex polyhydroxylated macrolide with a 40-membered ring, from a tropical marine cyanobacterium collected in the Palmyra Atoll. The compound showed potent antimalarial activity, with an IC_50_ of 223 nM against *P. falciparum* [119].

### 3.6. Extracts and Herbal Drugs

Medicinal plants have played a crucial role in the treatment of malaria and are often recommended as a strategy to combat resistance developed by *Plasmodium* parasites against conventional antimalarial drugs. Traditionally, herbal medicine has served as the foundation for the treatment of malaria for thousands of years.

The emergence of resistance in malaria parasites to conventional antimalarial drugs has renewed interest in herbal medicine as a potential alternative. Furthermore, the growing practice of combining herbal antimalarial therapies with conventional drugs, both synthetic and semisynthetic, has led researchers to investigate the interactions between these treatments. A comprehensive review article evaluated the outcomes of such combinations, highlighting synergistic, antagonistic, and neutral effects [69]. The results of studies involving extracts that showed additive effects when paired with conventional antimalarials indicate promising opportunities for the standardization and development of combination therapies that integrate medicinal plants with established antimalarial drugs.

This section presents only a few representative examples. Numerous studies have been conducted on the antimalarial effects of plant species used in traditional medicine across endemic regions, particularly in Africa. As previously noted, the main reasons for the use of plant extracts in malaria treatment include the emergence of parasite resistance to conventional antimalarial drugs, limited availability and accessibility of pharmaceuticals, and their high costs. In recent years, several comprehensive review articles have been published on plant species traditionally used for their ethnobotanical antimalarial properties [120,121,122,123,124]. This section aims to underscore the urgent need for the development of standardized protocols that address botanical identification, chemical content, safety, efficacy, and pharmacological properties.

For example, a study selected 13 Rwandan medicinal plants used to treat malaria based on ethnobotanical data and subjected the extracts to in vitro evaluation for their antiplasmodial and cytotoxic activities [125]. Most of the plants analyzed showed antiplasmodial activity (IC_50_ < 15 mg/mL) against chloroquine-resistant and sensitive *P. falciparum* strains. The best results were reported for the dichloromethane extracts of *Tithonia diversifolia*, *Microglossa pyrifolia*, *Rumex abyssinicus*, *Fuerstia africana*, *Zanthoxylum chalybeum,* and *Terminalia mollis*, the latter two being the ones that presented the best SI.

**Figure 13:**
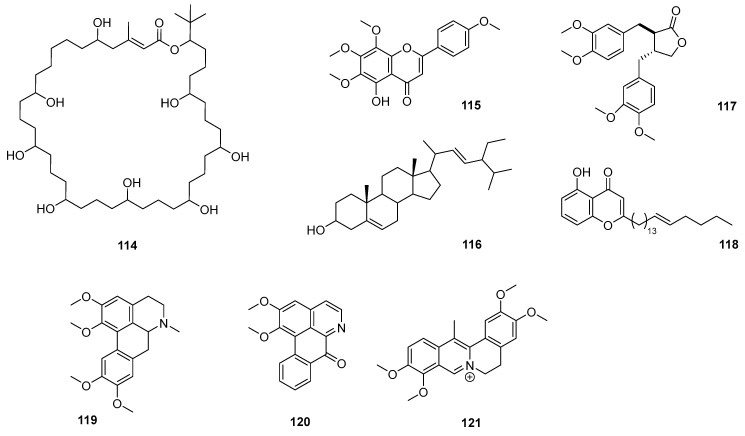
Antimalarial marine macrolide and natural compounds isolated from vegetal extracts.

In a recent study, *Citrus maxima* and *Artemisia nilagirica* were selected based on their traditional use in the treatment of fever associated with malaria in Assam, India. The most polar extracts were found to be the most powerful and effective (without being cytotoxic), showing IC_50_ values below 5 μg/mL against chloroquine-resistant and -sensitive strains. Preliminary qualitative studies revealed the presence of alkaloids, flavonoids, and terpenoids [126]. A 2014 study described the antiplasmodial activity of crude extracts, fractions, and pure compounds of *Peperomia vulcanica* and *P. fernandopoioana* (Piperaceae) through a bioguided test on chloroquine-sensitive and resistant strains of *P. falciparum*. Although the crude extracts of both species showed moderate activity, several of the compounds isolated from *P. vulcanica* in their pure forms were active and nontoxic. Four of them were characterized as 5-demethyltangeretin (**115**), stigmasterol (**116**), matairesinol dimethyl ether (**117**), and peperovulcanone A (**118**) (Figure 13) [127].

Another approach focuses on the evaluation of the antimalarial activity of a polyherbal antimalarial mixture compared to that of its individual components, supporting the use of combined therapies [128]. The polyherbal antimalarial mixture consisted of *Mangifera indica*, *Azadirachta indica*, *Nauclea latifolia*, and *Morinda lucida*. The results of this study revealed that polyherbal antimalarial mixture produced higher activity than its individual constituent plants (which also produced considerable parasite suppression) in both suppressive and prophylactic models, suggesting that the combination produced synergistic or additive effects. Considering these results, the authors recommend further studies to evaluate its clinical utility, safety, disposition, and interactions in humans.

Due to the intensive use of whole plants or plant mixtures instead of isolated compounds against this disease, it is important to note that there is evidence that crude plant extracts often have higher antiplasmodial activity in vitro or in vivothan isolated components at an equivalent dose. For example, these positive interactions denote a pharmacodynamic synergy between *Cinchona* alkaloids and various traditionally combined plant extracts. Pharmacokinetic interactions also occur between the components of *Artemisia annua* tea, so that ART is absorbed more quickly than the pure drug [129]. Something that is often observed for extracts is the immunomodulatory effect in addition to the direct antiplasmodial action. Plant-derived components can also be added to mitigate the side effects of others, for example, ginger to prevent nausea. While activity and efficacy can be improved, more clinical research is needed on all types of interactions between plant components.

Another type of study that needs to be performed in this area is the evaluation of the pharmaceutical and antimalarial quality of commercial formulations. An example is SAABMAL^®^, a polyherbal remedy used in Nigeria against malaria. Although the results obtained confirm the efficacy and quality of formulation of herbal antimalarial capsules, for these cases, it is necessary to develop appropriate analytical techniques capable of monitoring the release of active principles of the formulation, together with the establishment of stability and bioavailability parameters [130].

Regarding the tropical American area, in Brazil, a total of 33 extracts of 11 different species of the Atlantic Forest biome (*Mata Atlântica*) were evaluated against chloroquine-resistant *Plasmodium*. The results made it possible to identify *Alchornea glandulosa*, *Miconia latecrenata*, and *Psychotria suterella* as the most active species. Their bioguided fractionation led to the identification of different flavonoids and tannins in *A. glandulosa* and *M. latecrenata*. For *P. suterella*, alkaloids were identified, most of which had not been studied as antimalarials [131].

An interesting work postulates the standardization of the ethanolic extract of leaves of the *Annickia polycarpa* species, which has remarkable antimalarial activity with elimination of hyperparasitemia in mice infected with *P. berghei* [132]. At the same time, the extract was shown to be erythropoietic and immunomodulatory, with a high capacity to cancel deleterious hematological alterations produced by malaria, extending life and promoting the cure of mice. This activity is due to the biologically active aporphine and oxoaporphine [(glaucine (**119**), dehydrocorydaline (**120**), and lysicamine (**121**)]. Furthermore, the leaves of the *Annickia polycarpa* species exhibited a median lethal concentration (LC_50_) greater than 5000 mg/kg. With these results, the authors propose the isolation and characterization of components of the *Annickia polycarpa* species.

## 4. Phytonanotechnology to Beat Neglected Parasitosis

Since ancient times, plant-derived extracts and their bioactive constituents have been widely used worldwide for the prevention and treatment of human diseases [133]. However, the formulation of many phytochemicals remains challenging due to their inherently low bioavailability, poor water solubility, limited gastrointestinal absorption, and susceptibility to degradation [14,134]. To overcome these limitations, there has been growing interest in the development of phytonanoparticles/plant-derived nanoparticles (NPs), due to their biocompatibility, cost-effectiveness, low toxicity, and environmentally friendly characteristics [17]. Numerous studies have proposed the integration of phytochemical compounds into nanocarrier-based drug delivery systems as a promising strategy to improve pharmacokinetic and pharmacodynamic properties [15].

The application of nanotechnology is particularly advantageous, as nanostructured colloidal delivery systems can significantly enhance the solubility, chemical stability, and therapeutic efficacy of encapsulated bioactives, while also modifying their biodistribution [16]. Furthermore, nanoencapsulation offers the potential to reduce systemic toxicity and enable controlled or sustained drug release, further supporting the integration of nanocarriers in modern phytopharmaceutical formulations [17].

**Figure 14:**
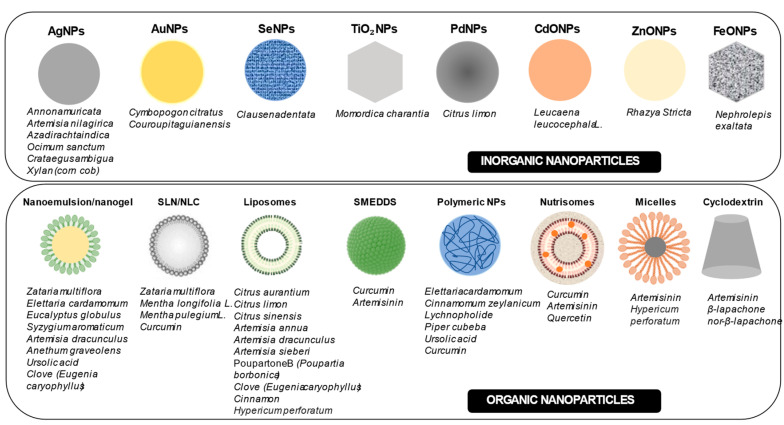
Schematic representation of various inorganic and organic nanocarriers synthesized using plant extracts or phytocompounds for potential application in phytotherapeutic delivery. Inorganic nanoparticles include silver (AgNPs), gold (AuNPs), selenium (SeNPs), titanium dioxide (TiO2NPs), palladium (PdNPs), cadmium oxide (CdONPs), zinc oxide (ZnONPs), and iron oxide (FeONPs), with different plant sources indicated. Organic nanocarriers include nanoemulsions, solid lipid nanoparticles (SLNs), nanostructured lipid carriers (NLCs), liposomes, self-microemulsifying drug delivery systems (SMEDDS), polymeric nanoparticles, nutrismos, micelles, and cyclodextrins, also based on various medicinal plants.

Nanostructures such as metallic NPs, polymeric NPs, lipid-based systems, and nanoemulsions are the most widely used for the incorporation of PNOs, with potential applications in neglected parasitic diseases such as malaria and Chagas. Figure 14 provides a comprehensive overview of the various nanocarriers, both inorganic and organic, that have been investigated for the delivery of plant-derived compounds. These nanosystems have shown great potential to enhance the therapeutic efficacy and bioavailability of phytochemicals.

Although much of the current research has focused on malaria, these nanocarriers also hold promise for the treatment of Chagas disease. However, studies targeting this application are still scarce and require further exploration.

### 4.1. Nanocarriers for Anti-Malarial Phytochemicals

Emerging approaches that use green nanotechnology offer a promising path for controlling malaria (see Table 1), as they can target and eliminate malaria vectors (*Anopheles* species) at both larval and adult mosquito stages, while also enabling the encapsulation and targeted delivery of current and novel antimalarial agents [135].

**Figure 15:**
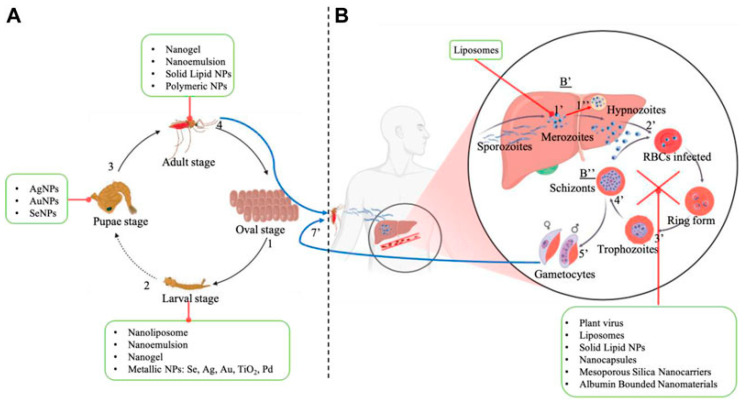
Schematic illustration of the life cycle of the Anopheles mosquito (**A**) and transmission cycle of plasmodium parasites (**B**). The life cycle of the Anopheles mosquito is divided into 4 stages: the female lays eggs in swampy areas (1), these eggs develop into larvae (2) within a few days, these larvae grow and mutate into pupae (3), which mature into adult mosquitoes (4). To ensure its survival, the hematophagous Anopheles feeds on human blood, and during this blood meal, the Anopheles, a vector of Plasmodium, infects humans and conducts the transmission cycle of plasmodium in human beings. Plasmodium parasites in humans develop in two phases: hepatocytes (B′) and erythrocytes (B″) stages. In the first stage (B′), the parasite undergoes perpetual mutation from sporozoites to merozoites (1′) in liver cells, and particularly for P. ovale and P. vivax, merozoites can enter a period of snooze, forming hypnozoites (1″). This phase is usually asymptomatic and can last from a few hours to a few days for P. falciparum and several days for P. ovale and P. vivax. When sufficiently colonized, hepatocytes are lysed and merozoites are released into the bloodstream, then start infection of erythrocytes (B″). This phase begins with merozoites invading RBCs (2″) and transforming into trophozoites (3″), then into schizonts (4″), which infect other RBCs. At the end of this phase, trophozoites differentiate into male and female gametocytes (5″), which are consumed by non-infected mosquitoes (7″) The gametocytes that infect the female mosquitoes undergo several mutations in the digestive tract, before finally transforming into sporozoites that migrate to the mosquito’s salivary glands and are injected into humans during the mosquito’s next blood meal. In this way, malaria spreads from person to person via the various mutations in humans and Anopheles mosquitoes. Reproduced with permission from [17] Creative Commons Attribution License (CC BY).

NPs play a crucial role in both the prevention and treatment of malaria, as shown in Figure 15 [17]. On the preventive side, they are employed in mosquito management strategies, such as the development of repellents and systems designed to eliminate larvae. On the therapeutic side, NPs are used to deliver antimalarial compounds more effectively against *Plasmodium falciparum*. This combined strategy takes advantage of the distinct functionalities of NPs to strengthen vector control efforts while enhancing the effectiveness of disease treatments [136].

The following section offers an in-depth overview of how plant-derived materials are used in two primary ways: as affordable sources for producing NPs with inherent biological activity, and as antimalarial substances, including plant-based extracts, fractions, and isolated compounds loaded in nanocarriers.

#### 4.1.1. Inorganic Nanocarriers

Inorganic NPs composed of metals such as silver, gold, zinc, iron, and selenium, either synthesized using plant extracts primarily as reducing and capping agents or loaded with phytochemicals, have garnered increasing scientific interest due to their potential applications in the management of parasitic diseases such as malaria. These nanosystems have been investigated both as insect repellents and as therapeutic agents for human use.

The use of gold nanoparticles (AuNPs) synthesized from the leaf extract of *Cymbopogon citratus* has been investigated for their larvicidal and pupicidal activity against *An. Stephensi*. NPs with a size distribution in the range of 20–50 nm can be used at very low concentrations to enhance the control of larval *Anopheles* populations in copepod-based control programs [137]. Other authors explored the use of *Couroupita guianensis* flower extract as a reducing and stabilizing agent for the biosynthesis of AuNPs. The resulting AuNPs, with similar sizes to those previously described (29.2–43.8 nm), demonstrated significant toxicity against *An. stephensi* larvae, pupae, and adults, with lower IC_50_ values than the crude flower extract. The enhanced mosquitocidal potential of the AuNPs compared to the extract alone was attributed to their polydispersity and high stability in water, which allows them to persist for several weeks. This stability facilitates their penetration through the insect cuticle and even into individual cells, where they interfere with molting and other vital physiological processes. In addition to their larvicidal activity, the antiplasmodial potential of both the *C. guianensis* flower extract and the synthesized AuNPs was evaluated against chloroquine-sensitive and resistant strains of *P. falciparum*. Both treatments exhibited greater activity than chloroquine, indicating promising dual efficacy as mosquitocidal and antiplasmodial agents [138].

In addition, silver nanoparticles (AgNPs) synthesized using different medicinal plants have been widely explored by various authors. For instance, Santhosh et al. [139] synthesized AgNPs using *Annona muricata* leaf extract and demonstrated that they induced higher mortality in *An. stephensi* compared to the aqueous leaf extract. While the *A. muricata* aqueous leaf extract displayed LC_50_ and LC_90_ values of 61.38 and 156.55 μg/mL against third-instar *An. stephensi*, AgNPs showed lower LC_50_ and LC_90_ values of 15.28 and 31.91 μg/mL, respectively, indicating enhanced efficacy.

Nalini et al. [140] synthesized AgNPs using the aqueous leaf filtrate of *Artemisia nilagirica* as a potential mosquito control strategy. The bioefficacy of these phyto-synthesized AgNPs demonstrated significantly increased larvicidal and pupicidal activity against different stages (instars I–IV and pupae) of the malaria vector, compared to the activity observed with the aqueous leaf extract alone. The pupal stage showed the highest sensitivity to AgNP treatment, with an LC_50_ value of 0.050%, followed by fourth- to first-instar larvae, with LC_50_ values ranging from 0.141% to 0.343%. In contrast, treatment with the aqueous leaf extract alone resulted in approximately a 2-fold higher LC_50_ value, ranging from 0.066% to 0.722% across the same developmental stages. The exact mechanism of action of AgNPs remains unclear; however, it is proposed that these NPs can penetrate the larval membrane by interacting with cellular components, ultimately leading to larval death. Once AgNPs reach the midgut epithelial membrane, they appear to inactivate key enzymes and promote peroxide generation, triggering cell death. Ojemaye et al. synthesized and characterized AgNPs mediated by aqueous fruit and leaf extracts of *Crataegus ambigua* and investigated their antimalarial efficacy against *P. falciparum* parasites, showing excellent activity with 100% inhibition at 20 μg/mL, compared to 48.5% for the plant extract [141].

*Azadirachta indica* (neem) is widely recognized for its broad spectrum of biological activities. Its leaves, seeds, and bark are rich in bioactive compounds such as azadirachtin, nimbin, flavonoids, and terpenoids, which exhibit antimicrobial, antiparasitic, antifungal, and insecticidal properties. Due to these characteristics, it has been extensively used as a natural source for the green synthesis of metal NPs, particularly AgNPs, where it serves as a reducing, capping, and stabilizing agent [142].

Murugan et al. [143] developed AgNPs using the *Azadirachta indica* seed kernel, which demonstrated promising antiparasitic activity at multiple biological levels. Although the seed kernel extract exhibited larvicidal and pupicidal activity against *An. stephensi*, with LC_50_ values ranging from 232.8 ppm (first instar) to 348.4 ppm (pupal stage), the AgNPs showed markedly lower LC_50_ values (3.9 ppm (first instar) and 8.2 ppm (pupal stage)), highlighting their enhanced efficacy. Furthermore, in vitro antiplasmodial assays indicated that AgNPs had slightly greater activity than the extract, and in vivo studies in albino mice supported these findings. Despite these results, their most pronounced effect was as mosquito repellents, underscoring the versatility of *A. indica*-derived AgNPs for integrated malaria control strategies.

Sardana et al. also synthesized and characterized AgNPs using different compositions of aqueous leaf extracts of *Azadirachta indica* and *Ocimum sanctum* (tulsi), another plant with recognized antimalarial and antiplasmodial activity [144]. The authors obtained NPs with sizes ranging from 4.74 to 39.32 nm and with EC_50_ values between 0.313 and 1.692 μM in vitro against the *P. falciparum* 3D7 strain. NPs containing only *Azadirachta indica* or a combination of *A. indica–O. sanctum* (80:20) showed the best efficacy, with the lowest EC_50_ values, representing a multiple increase in antiplasmodial activity compared to pure leaf extracts. This improvement was attributed to the small size and spherical shape of the AgNPs, which allowed greater penetration into parasites [145].

More recently, Hawadak et al. [146] synthesized and characterized spheroidal, crystalline, and stable AgNPs from aqueous leaf and bark extract of *A. indica*, with a mean size of 13.01–19.30 nm. These demonstrated good antiplasmodial activity against both sensitive (3D7) and resistant (RKL9) strains of *P. falciparum*, with IC_50_ values of 9.27 and 11.14 μg/mL for leaf-derived NPs, and 8.10 and 7.87 μg/mL for bark-derived NPs, respectively. These values were significantly lower than the IC_50_ values of the crude extracts: 33.97 and 49.64 μg/mL for the leaf and bark extracts against 3D7, and 46.82 and 54.10 μg/mL against RKL9. However, significant hemolytic activity (>25%), indicative of membrane damage, was observed for both AgNPs at 125 μg/mL. This effect is attributed to the small particle size and negative surface charge of AgNPs, consistent with previous reports [147]. More studies are needed to optimize the synthesis parameters and surface modifications of AgNPs to improve their hemocompatibility and reduce potential cytotoxic effects.

Although AgNPs and AuNPs have been extensively studied with various phytochemicals for malaria treatment, other metallic NPs have also been explored, including Se, TiO_2_, Pd, ZnO, CdO, FeO NPs, with promising antimalarial activity.

For example, selenium NPs (SeNPs) were synthesized using *Clausena dentata*, and their larvicidal activity against *An. stephensi* larvae was evaluated. SeNPs (46.32–78.88 nm) exhibited a very low LC_50_ (240.714 mg/L) compared to the aqueous leaf extract (313.116 mg/L), indicating high toxicity to mosquito larvae without adverse effects on nontarget aquatic organisms [148]. Their larvicidal effects were attributed to oxidative stress induction, leading to cell death via reactive oxygen species generation [149].

Titanium dioxide NPs (TiO_2_ NPs) synthesized using aqueous leaf extract of *Momordica charantia* were also investigated. These NPs showed significantly enhanced bioactivity compared to the crude extract. TiO_2_ NPs exhibited high toxicity against *An. stephensi* at low concentrations. LC_50_ values for the extract ranged from 53.29 to 96.09 mg/L across various larval stages and pupae, whereas TiO_2_ NPs ranged from 2.50 to 5.04 mg/L. In vitro assays confirmed that *M. charantia*-mediated TiO_2_ NPs were more effective than extract alone. IC_50_ values were 53.42 μg/mL (chloroquine-sensitive) and 59.71 μg/mL (chloroquine-resistant), versus 83.64 μg/mL (chloroquine-sensitive) and 88.14 μg/mL (chloroquine-resistant) for the extract [149].

Green synthesis of ZnO NPs using *Rhazya stricta* leaf extract yielded particles averaging 70–90 nm. In vitro antiplasmodial activity test showed greater efficacy for the NPs (IC_50_ = 3.41 5 μg/mL) versus extract alone (IC_50_ = 19.3 μg/mL), with good biocompatibility up to 2.5 mg based on hemolysis data [150].

Iron oxide NPs (FeO NPs) synthesized using *Nephrolepis exaltata* extract were spherical (~16 nm), crystalline, and demonstrated significantly higher parasite inhibition (62 ± 1.3% at 25 µg/mL) compared to the extract (35%) and FeCl_3_6H_2_O precursor (23%). No cytotoxic effects were observed in human peripheral blood mononuclear cells [151]. A potential synthesis mechanism involves catechins in the extract acting as reducing and stabilizing agents. The high density of phenolic hydroxyl groups facilitates redox reactions, reducing iron salts and stabilizing FeO NPs via chelation. H^+^ radicals generated from electron donation further support Fe ion reduction [152].

In general, the synthesis of inorganic NPs using plant extracts as reducing and stabilizing agents represents a promising green nanotechnological approach for vector and malaria control. Studies involving *Azadirachta indica*, *Annona muricata*, *Clausena dentata*, and others consistently demonstrate that NP formulations exhibit significantly enhanced larvicidal and antiplasmodial activities compared to crude extracts. This increased efficacy is largely attributed to the physicochemical properties, including aqueous stability, cuticles, and cell penetration, and the induction of oxidative stress, making them eco-friendly, effective, and sustainable alternatives to conventional malaria control strategies.

#### 4.1.2. Organic Nanocarriers

##### Polymer-Based NPs

Polymer-based NPs have emerged as a versatile platform for the delivery of bioactive compounds, including phytochemicals with antimalarial potential. These systems offer several advantages, such as controlled release, protection of labile molecules from degradation, and the possibility of surface functionalization for targeted delivery. Natural and synthetic polymers, including poly(D,L-lactic-co-glycolic acid) (PLGA), chitosan, and polyethylene glycol (PEG)-based materials, have been widely used to encapsulate plant-derived compounds such as curcumin, quercetin, and ART [153]. For instance, curcumin and artesunate, both known for their antiplasmodial activities, were co-encapsulated in PLGA NPs using the solvent evaporation method from oil-in-water single emulsion. This formulation, with a mean particle size of (251.1 ± 12.6) nm and an entrapment efficiency of (22.3 ± 0.4)%, demonstrated sustained drug release over seven days and achieved significant suppression of parasites in murine models, with a reduction of more than 70% in parasitemia at lower doses compared to free drugs. Furthermore, the PLGA-based system exhibited favorable safety profiles, indicating its potential for malaria therapy [154].

Similarly, PLGA-curcumin NPs were obtained using a single emulsion solvent evaporation method, with a superior therapeutic index compared to native curcumin. These NPs demonstrated greater effectiveness in preventing the onset of neurological symptoms and prolonging survival in a *P. berghei*-infected mouse model of experimental cerebral malaria [155]. In another study, PLGA-curcumin NPs synthesized using the same technique demonstrated enhanced antimalarial potency compared to free (nonencapsulated) curcumin. Moreover, the NPs exhibited improved efficacy at lower concentrations, offering the advantage of minimizing high-dose drug-related toxicity. In vivotoxicity assessments further confirmed the safety of formulated NPs at the doses tested [156].

Polymer-based NPs were investigated as repellents. For example, essential oils from *Elettaria cardamomum* and *Cinnamomum zeylanicum* were characterized and encapsulated in chitosan NPs by ionic gelation. Although their repellent activity ranged from 3 to 34 min (less than N,N-diethyl-meta-toluamide (DEET) (62 ± 5) min), the nanoformulations demonstrated strong larvicidal effects against *An. stephensi*, achieving 100% mortality at 25 µg/mL. Furthermore, both formulations were not cytotoxic to normal human skin cells (HFFF2), highlighting their potential as safe, plant-based larvicides [157].

##### Nanoemulsions and Nanogels

Nanoemulsions, which are thermodynamically unstable but kinetically stable colloidal dispersions composed of two immiscible liquids (typically oil and water) and stabilized by surfactants, offer advantages such as enhanced solubility, increased surface area, and improved bioavailability of lipophilic compounds, including essential oils [158]. Similarly, nanogels are three-dimensional nanosized crosslinked polymer networks capable of holding large amounts of water or biological fluids [159]. Their unique properties, including high surface area, biocompatibility, and controlled release capabilities, make them suitable carriers for various bioactive agents. It is now widely recognized that incorporating essential oils into nanoemulsion and nanogel formulations enhances both their stability and the biological efficacy of plant-based mosquito repellents [160].

Nanoemulsion-based nanogels formulated with essential oils of *Zataria multiflora* and *Elettaria cardamomum* were evaluated for mosquito repellent activity against the primary malaria vector, *An. stephensi* [161]. The nanogel containing 2.5% essential oil of *Z. multiflora*, with a droplet size of 8  ±  1 nm, showed superior protection, with a complete protection time (CPT) of 600 min, significantly outperforming DEET (CPT: 242 min), likely due to its active compounds, carvacrol and thymol, which are known for their insect repellent properties. In contrast, the nanogel of *E. cardamomum* essential oil, with a droplet size of 86  ±  5 nm, showed significantly less efficacy than DEET, with a CPT of 63  ±  15 min.

In a similar study conducted by Sheikh et al. [162], the authors developed a nanoemulsion using essential oils from *Eucalyptus globulus* and *Syzygium aromaticum*, employing a low-energy emulsification method by magnetic stirrer. Protection times of textures impregnated with different bulk essential oils or nanoemulsions were measured against *An. Stephensi* mosquitoes. The CPT values of textiles treated with *E. globulus* (1%) and *S. aromaticum* (0.5%) were less than 2 min, while the nanoemulsion prepared with the most effective combination of essential oils showed the highest CPT (285 ± 30 min) compared to the others (<5 min). The authors attributed the longer CPT to the reduced evaporation rate of the volatile components of essential oils within the nanoemulsion formulation.

In nanoemulsions, essential oil is encapsulated within a layer of emulsifier molecules, which helps to limit the evaporation of active compounds, thereby promoting a slow and continuous release [158]. Furthermore, the small droplet size of the nanoemulsion contributes significantly to the extended protection time, since NPs are likely to penetrate various substrates, including textile fibers, more effectively [163]. Additionally, it is widely recognized that the nanoscale size of the droplets allows for more uniform spraying or deposition onto target surfaces, enhancing the overall efficacy of the formulation.

More recently, Osanloo et al. [164] prepared a nanoemulsion and a nanogel containing *Artemisia dracunculus* (Asteraceae) essential oil and evaluated the larvicidal effects against *An. Stephensi.* In this study, dose-dependent responses were observed in their efficacy, and the nanogel was more potent (IC_50_ = 6.6 μg/mL) than the nanoemulsion (IC_50_ = 13.5 μg/mL), a result attributed to its better stability and sustained release profile [164].

##### Solid Lipid Nanoparticles

Lipid-based NPs are versatile nanocarriers in drug delivery systems due to their biocompatibility, ability to encapsulate both hydrophilic and lipophilic compounds, and potential for controlled release [165]. This strategy has been explored for malaria, particularly for encapsulating essential oils as repellents. For example, solid lipid nanoparticles (SLNs) containing three essential oils (*Mentha longifolia* L., *Mentha pulegium* L., and *Zataria multiflora* Boiss.) were prepared using high-pressure homogenizer, and their larvicidal effects against *An. stephensi* were evaluated and compared. The sizes of the SLNs containing *Mentha longifolia*, *Mentha pulegium*, and *Zataria multiflora* essential oil were (105 ± 7), (210 ± 4), and (137 ± 8) nm, respectively; and their efficacy, with LC_50_ values of 24.79, 5.11, and 9.19 µg/mL, was significantly more potent than that of their essential oils, which had LC_50_ values of 36.2, 27.55, and 33.33 µg/mL, with the most promising system being the SLNs containing *M. pulegium* [166]. *Zataria multiflora* essential oil was encapsulated in another SLN using the same method, with a similar size of 134 ± 7 nm, polydispersity index (PDI) of 0.24 ± 0.1, and an entrapment efficiency of 64.6 ± 3.8%. The CPT of these SLNs was 93 ± 5 min, three times longer than that of the nonformulated essential oil (29 ± 2 min) [167].

##### Liposomes

Various studies have been conducted to develop liposomes as nanocarriers of essential oils. Sanei-Dehkordi et al. [168] reported the larvicidal effect of the three essential oils (*Citrus aurantium*, *Citrus limon*, *Citrus sinensis*) and limonene against *An. stephensi*, demonstrating that the larvicidal effect of nanoformulated forms was more effective than their nonformulated counterparts. Nanoliposomes containing *Citrus aurantium* essential oil showed the best larvicidal activity, suggesting its potential as an alternative to synthetic insecticides. Similarly, Sanei-Dehkordi et al. [169] employed the same platform but encapsulating essential oils of *Artemisia annua*, *Artemisia dracunculus*, and *Artemisia sieberi*, and revealed that nanoliposomes containing *A. dracunculus* showed the highest potential larvicidal effect against *An. stephensi*.

##### Nanostructured Lipid Carriers

Nanostructured lipid carriers (NLCs), regarded as the second generation of SLNs, have been developed with enhanced characteristics, including a less-ordered lipid matrix that enables more stable and prolonged encapsulation of active compounds [170]. These systems have also been explored for applications in malaria treatment. For example, curcumin-loaded NLCs were prepared using the microemulsion method, and their antiplasmodial activity was studied in mice infected with *Plasmodium berghei* [171]. The curcumin-loaded NLCs exhibited a mean particle size of 145 nm, a PDI of 0.3, and a zeta potential of −25 mV, with pseudospherical morphology confirmed by transmission electron microscopy (TEM) analysis. The formulation achieved a loading capacity of 3.1% and an encapsulation efficiency of 74%. In vitro studies revealed that curcumin release from NLCs followed a sustained-release profile over five days, and in vivo studies demonstrated that curcumin NLCs significantly enhanced antiplasmodial activity compared to free curcumin at 40 mg/kg/day.

##### Nutriosomes

Nutriosomes, phospholipid vesicles associated with Nutriose^®^ FM06, a soluble dextrin with prebiotic activity suitable for oral delivery, were also explored for the treatment of malaria. ART, curcumin, or quercetin, either alone or in combination, were loaded into this nanocarrier, achieving sizes between 93 and 146 nm. The authors demonstrated a sustained release profile for curcumin and quercetin from nutriosomes, with a release of approximately 53% observed after 48 h, while ART exhibited a rapid release, reaching nearly 100% within the same period. Cytotoxicity assays carried out on human colon adenocarcinoma cells (Caco-2) and human umbilical vein endothelial cells confirmed the high biocompatibility of the developed formulations. Furthermore, in vitro antimalarial activity assays against the 3D7 strain of *Plasmodium falciparum* validated the potential of nutriosomes for the effective delivery of curcumin and quercetin as adjuvants in antimalarial therapy. Although the efficacy of ART was confirmed, no enhancement in its activity was observed [172].

The nutrisomes were also modified by the addition of Eudragit^®^ to obtain systems aimed at enhancing the efficacy of curcumin in counteracting malaria infection upon oral administration. Eudragit nutriosomes demonstrated promising in vitro and in vivo performance, significantly enhancing curcumin’s ability to mitigate oxidative stress in intestinal Caco-2 cells, which likely contributed to improved systemic efficacy. Cryogenic TEM analysis revealed the presence of multicompartment vesicles with an average diameter of approximately 300 nm and a strongly negative zeta potential. The vesicles exhibited high stability in simulated gastrointestinal fluids, attributed to the elevated phospholipid content, as well as the protective effects of gastro-resistant Eudragit and digestion-resistant Nutriose [173].

##### Self-Microemulsifying Drug Delivery Systems

Self-microemulsifying drug delivery systems (SMEDDS) have gained significant attention as an effective strategy to enhance the solubility, dissolution rate, and oral bioavailability of poorly water-soluble drugs. SMEDDS consist of an isotropic mixture of oil, surfactant, cosurfactant, and the drug, which spontaneously forms a microemulsion upon exposure to gastrointestinal fluids and motility following oral administration. The resulting microemulsion, typically with droplet sizes below 100 nm, improves the solubility of hydrophobic compounds and promotes their absorption throughout the gastrointestinal tract [174].

SMEDDS have been developed to enhance the solubility of curcumin and ART [175]. The optimal formulation was obtained using a D-optimal mixture design, resulting in an average particle size of 150.7 nm. The system was thoroughly characterized, and solubility enhancement was clearly demonstrated. In a diffusion study, more than 63.8% of curcumin and 54.9% of ART were dissolved in a pH 1.2 medium within 90 min. However, this study did not evaluate the efficacy or safety of the new SMEDDS formulation containing curcumin and ART. Future research could explore not only the performance of the nanocarrier but also the potential synergistic antimalarial effects of both phytochemicals in vitro and in vivo.

##### Micelles and Cyclodextrins

Micelles and cyclodextrins (CDs) are prominent nanocarriers extensively studied for their roles in enhancing the solubility, stability, and bioavailability of poorly water-soluble drugs. Micelles, formed by the self-assembly of amphiphilic molecules in aqueous media, provide a hydrophobic core that can encapsulate lipophilic compounds, while their hydrophilic shell ensures colloidal stability [176]. CDs, on the other hand, are cyclic oligosaccharides that are known to form inclusion complexes with a wide range of drug molecules, thereby enhancing their aqueous solubility and protecting them from degradation. Recent studies have highlighted the versatility of CDs in pharmaceutical formulations, noting their role in improving drug solubility, stability, and bioavailability [177]. Both nanocarriers have been explored for the delivery of ART. This compound was successfully encapsulated within mPEG-PCL micelles with a single-step nanoprecipitation method, obtaining a spherical shape and a mean size ranging between 110.34 and 142.9 nm, measured by atomic force microscopy and dynamic light scattering [178]. The encapsulation efficiency was (63 ± 2.31)%, and the loading efficiency of ART-loaded copolymeric micelles was (15 ± 1.43)%. In vitro release of ART from micelles followed a remarkably sustained release profile. Although this work was not specifically focused on malaria, its evaluation in this context would be of great interest, as the authors successfully developed a system using a simple methodology that encapsulates a phytochemical with well-documented antimalarial activity and allows its sustained release.

ART was also successfully encapsulated in CD-based nanocarriers, with a specific focus on malaria treatment. Surface-decorated amphiphilic γ-CD NPs were engineered using a co-nanoprecipitation method with bio-esterified CDs and PEG derivatives such as polysorbate 80 and DMPE-mPEG2000. Two types of formulations: nanospheres with a mean size of 92 nm (PDI = 0.15) and nanoreservoirs with a size of 188 nm (PDI = 0.06) were developed, characterized, and evaluated in vivo in healthy rats after a single intravenous dose (1.5 or 2 mg/kg). Both nanosuspensions had a stable ART loading, corresponding to drug levels reaching 0.48 and 1.65 mg/mL for nanospheres and nanoreservoirs, respectively. The drug recovery corresponding to the amount of ART in the nanosuspensions was around 95%, and the drug association was 88% for nanospheres and 96% for nanoreservoir systems. In comparison with an ethanolic-aqueous ART solution, both nanoformulations significantly improved the pharmacokinetic profile of the drug. In particular, ART-loaded nanospheres and nanoreservoirs increased plasma exposure (AUC_0_–t) by 3.26- and 2.35-fold, respectively, while extending their half-life by up to 6.25-fold and reducing clearance by as much as 4.72-fold [179].

**Table 1:** Summary of nanocarrier systems incorporating anti-malarial phytochemicals.

Nanocarrier	Nanocarrier Composition	Plant (Part/s Used)	Main Chemical Constituents	Size (nm)	Stage of Development	Reference
Inorganic NPs	AuNPs	*Cymbopogon citratus*	Volatile oils, flavonoids, phenolic acids, phenylpropanoids, alcohols, esters, aldehydes, and alkaloids	20–50	In vivo (larvae and pupae of *An. stephensi*)	[137]
	AuNPs	*Couroupita guianensis* (flowers)	Flavonoids, phenolics, alkaloids	29–44	In vivo (larvae, pupae, and adults of *An. stephensi*)	[138]
	AgNPs	*Annona muricata* (leaves)	Acetogenins, flavonoids, alkaloids	20–53	In vivo(larvae *An. stephensi)*	[139]
	AgNPs	*Azadirachta indica* (leaves and bark)	Azadirachtin, nimbin, flavonoids, and terpenoids	<20	In vitro (3D7 and RKL9 *P. falciparum* strains)	[146]
	AgNPs	*Azadirachta indica*		35–60	In vivo(albino mice)	[143]
	AgNPs	*Azadirachta indica* and *Ocimum sanctum*	Phenols, terpenoids	<40	In vitro (*P. falciparum* 3D7 parasites)	[145]
	AgNPs	*Artemisia nilagirica* (leaves)	ART, quercetin, apigenin, Β-caryophyllene, luteolin, and simple phenolic acids	<30	In vivo(larvae I–IV instar, and pupa of *An. stephensi*)	[140]
	AgNPs	*Crataegus* *ambigua* (leaves and fruit)	Oligomeric procyanidins, flavonoids, triterpenes, polysaccharides, catecholamines	30	In vitro (NF54 strain of *P. falciparum* parasites)	[141]
	CdONPs	*Leucaena leucocephala* L. (aqueous plant extract)	Tannins, saponins, amino acids, steroids, flavonoids, phenols, carbohydrates, coumarins, cardiac glycoside	36–57	In vitro (*P. falciparum*)	[180]
	ZnONPs	*Rhazya stricta* (leaves extract)	Tannins, saponins, amino acids, gallic acid, and various flavonoids	19	In vitro (*P. falciparum*)	[150]
	FeONPs	*Nephrolepis exaltata*	Saponins, steroids, alkaloids, phenols, and tannins (notably catechin)	16	In vitro (*P. falciparum*)	[151]
	SeNPs	*Clausena dentata* (leaves)	Coumarins, alkaloids, steroids, terpenoids, essential oils, and flavonoids	46–79	In vivo(larvae I–IV instar of *An. stephensi*)	[148]
	TiO_2_NPs	*Momordica charantia* (leaves)	Alkaloids, saponins, glycosides, phenolic constituents, reducing sugars and free acids	35–70	In vivo (larvae I–IV instar, and pupa of *An. stephensi*)	[181]
	PdNPs	*Citrus limon* (leaves)	Essential oils (limonene), flavonoids, phenolic acid	2–4.8	In vivo (3rd instar larvae of *An. stephensi*)	[182]
Nanoemulsion/nanogels	Tween^®^ 20 and carboxymethylcellulose	*Elettaria cardamomum* (leaves)	Essential oils (1,8-cineole, terpineol, limonene, terpinyl acetates, linalyl acetate, linalool, sabinene)	86	In vivo (larvae *An. stephensi*)	[161]
	Tween^®^ 20 and carboxymethylcellulose	*Zataria multiflora* (leaves)	Essential oils (Thymol and cavacrol)	8	In vivo (larvae *An. stephensi*)	
	Tween 80^®^, Tween 20^®^, propylene glycol	*Eucalyptus globulus* and *Syzygium aromaticum*	Essential oils (1,8-cineole and eugenol)	11–23	In vivo (larvae *An. stephensi*)	[162]
	Tween 20^®^	*Anethum graveolens*	Essential oils (alpha-Phellandrene, p-Cymene, Carvone)	<20	In vivo (larvae *An. stephensi*)	[183]
	Tween^®^ 20	*Artemisia dracunculus*	Essential oils (estragole, sabinene, methyl eugenol, elemicin)	152	In vivo (late-3rd or young-4th instar larvae *An. stephensi)*	[164]
	Tween^®^ 20, carboxymethylcellulose	*Artemisia dracunculus*		NA		
SLNs	Stearic acid/Span^®^ 60/Tween^®^ 80	*Zataria* *multiflora*	Essential oils (thymol and cavacrol)	134	In vivo (larvae *An. stephensi*)	[167]
	Stearic acid/Span^®^ 60/Tween^®^ 80	*Mentha longifolia*	Essential oils (pulegone, 1,8-cineole, piperitone, menthone)	105	In vivo (larvae *An. stephensi*)	[166]
		*Mentha pulegium*	Essential oils (pulegone, piperitone, limonene)	210		
		*Zataria* *multiflora*	Essential oils (thymol and cavacrol)	137		
Nanostructured lipid carriers (NLC)	Coconut oil and cetyl palmitate	*Curcuma longa* (rhizomes)	Curcumin	145	In vivo (mice)	[171]
Nanoliposomes	Lecithin, cholesterol, Tween^®^ 20	*Citrus* *aurantium*	Essential oils (limonene)	42–67	In vivo (larvae *An. stephensi*)	[168]
		*Citrus limon*				
		*Citrus sinensis*				
	lecithin, cholesterol, Tween^®^ 20	*Artemisia* *annua*	Essential oils (ART, camphor, 1,8-cineole)	137	In vivo (larvae *An. stephensi*)	[169]
		*Artemisia* *dracunculus*	Essential oils (estragole, cis-ocimene, ɣ-terpinene)	151		
		*Artemisia* *sieberi*	Essential oils (camphor, α/β-thujone)	92		
	Heparin, DOTAP, DOPC, cholesterol	*Poupartia borbonica*	Poupartone B	183–256	In vitro (*P. falciparum*)	[184]
	Lecithin, cholesterol, Tween^®^ 20	*Syzygium aromaticum*	Essential oils (eugenol)	109–158	In vivo (larvae *An. stephensi*)	[185]
		*Cinnamomum zeylanicum*	Essential oils (cinnamaldehyde)	111–195		
Polymeric NPs	Chitosan/ Tween^®^ 20	*Elettaria* *cardamomum* and *Cinnamomum zeylanicum*	Essential oils (1,8-cineole and cinnamaldehyde)	78–204	In vivo (larvae *An. stephensi*)	[157]
	PLGA	*Curcuma longa* (rhizomes)	Curcumin	495	In vivo (mice)	[155]
	PLGA	*Curcuma longa* (rhizomes)	Curcumin	291	In vivo (mice)	[156]
	PLGA	*Curcuma longa* (rhizomes)	Curcumin	251	In vivo (mice)	[154]
Zwitterionic self-assembled NPs	PBMA- MESBMA	*Curcuma longa* (rhizomes)	Curcumin	20–100	In vivo (mice)	[186]
Nutrisomes	Nutriose FM06^®^, Eudragit^®^	*Curcuma longa* (rhizomes)	Curcumin	300–337	In vivo (mice)	[173]
	Nutriose FM06^®^, S75	*Curcuma longa* (rhizomes)	Curcumin	108–152	In vitro (*P. falciparum*)	[172]
		*Artemisia annua*	ART	93–95		
		NA	Quercetin (polyphenol)	121–125		
		*Artemisia annua* and *Curcuma longa*	ART-curcumin	111–121		
		*Curcuma longa*, NA	ART-quercetin	119–128		
Nanocapsules	PCL, MCT, Span 60^®^, and (coatings: P80, PEG, Chitosan, or Eudragit RS100^®^)	*Curcuma longa* (rhizomes)	Curcumin	123–250	In vitro	[187]
Micelles	mPEG-PCL	*Artemisia annua*	ART	110–143	In vitro	[178]
γ-CD NPs	Sorbitan monooleate, P80, DMPEmPEG2000	*Artemisia annua*	ART	92–188	In vivo (Wistar rats)	[179]

NA: not available.

### 4.2. Nanocarriers for Anti-Chagasic Phytochemicals

Chagas disease, caused by the protozoan *T. cruzi*, remains a significant public health concern, with limited effective treatments and considerable side effects associated with current therapies. Phytochemicals, such as UA, lychnopholide (LYC), and curcumin, have demonstrated antiparasitic and immunomodulatory properties. However, their clinical application is often hindered by poor solubility, low bioavailability, and rapid metabolism [188]. To address these challenges, different nanocarrier systems have been developed to encapsulate these bioactive compounds, improving their pharmacokinetic profiles and therapeutic outcomes (Table 2).

#### 4.2.1. Inorganic Nanocarriers

In contrast to the extensive research on inorganic NPs for malaria, there is a noticeable scarcity of studies addressing their use in the treatment of Chagas disease. In 2020, AgNPs were explored for the first time as a potential therapeutic approach against *T. cruzi*. Using xylan, a bioactive polysaccharide extracted from corn cobs, researchers successfully synthesized green, spherical-shaped AgNPs. These NPs, with a particle size of (55.3 ± 10) nm, demonstrated significant trypanocidal activity against the Y strain epimastigotes of *T. cruzi*, inducing parasite death, primarily through a necrotic mechanism. In particular, neither xylan nor Ag alone exhibited such effects, underscoring the importance of the NP formulation for biological efficacy [189]. The findings suggest that nanoxylans have potential as a therapeutic alternative for the treatment of Chagas disease, warranting further validation through future in vivo studies.

#### 4.2.2. Organic Nanocarriers

##### Polymer-Based NPs

Several studies have reported the use of phytochemicals with trypanocidal activity encapsulated in different nanocarrier systems to overcome their pharmacokinetic limitations and improve their therapeutic efficacy. Among these, LYC, a bioactive compound (specifically an SL derived from plants of the *Asteraceae* family), exhibits strong trypanocidal activity. However, its therapeutic potential is hindered by poor water solubility and high lipophilicity. To address these drawbacks, de Mello et al. [190] explored the use of polymeric nanocapsules to encapsulate LYC in an infected murine model. LYC was loaded into conventional nanocapsules with poly-ɛ-caprolactone (PCL) and sterically stabilized nanocapsules with poly(D,L-lactide) acid (PLA) and PEG, via interfacial deposition of the preformed polymer followed by solvent displacement. The mean hydrodynamic diameters were 190.2 ± 5.7 for PCL-nanocapsules and 106.1 ± 6.3 nm for PLA-PEG-nanocapsules, both with high drug loading of 95% and 100%, respectively, PDI < 0.3, and zeta potential values around −33 mV.

In a murine model infected with the partially benznidazole-resistant Y strain of *T. cruzi*, oral treatment with LYC-PLA-PEG nanocapsules led to superior outcomes compared to free LYC and other nanocapsule formulations. Mice treated with LYC-PLA-PEG nanocapsules during the acute phase exhibited complete survival and a cure rate of 62.5%, matching that of benznidazole and exceeding the results of conventional nanocapsules and free LYC. Notably, during the chronic phase, LYC-PLA-PEG nanocapsules achieved a cure rate of 55.6%, while other treatments, including free LYC and benznidazole, were ineffective. The improved efficacy of the PEG-containing nanocarriers is attributed to their ability to prolong systemic circulation, reduce opsonization, and provide protection against gastrointestinal degradation, allowing sustained drug release and better targeting of intracellular and interstitial parasite forms [191]. Intravenous administration was also evaluated during the acute phase, demonstrating anti-*T. cruzi* efficacy only with LYC-nanocapsules; no cure was observed for animals in the other groups (free LYC, benznidazole, and controls). This study represents the first report of successful oral treatment of experimental Chagas disease using an isolated phytochemical encapsulated in both conventional and sterically stabilized polymeric nanocapsules during both infection phases.

In a subsequent study, Branquinho et al. [192,193,194] conducted an in-depth investigation of the cardiotoxicity of LYC-loaded nanocapsules, as well as the pharmacokinetic profile and efficacy in mice infected with a *T. cruzi* strain completely resistant to drugs in both phases of infection, demonstrating that nanoencapsulation prevents the cardiac alterations caused in vivo by free LYC, improves the pharmacokinetic parameters, and leads to complete cure in mice treated with the highest dose of loaded nanocapsules. More specifically, the authors demonstrated that the cardiotoxicity observed during repeated-dose treatment with LYC in its free form was mitigated when LYC was encapsulated in polymeric nanocapsules (PLA-PEG), as evaluated in healthy C57BL/6 mice. Regarding pharmacokinetics in female Swiss albino mice, LYC-PLC nanocapsules and LYC-PLA-PEG nanocapsules showed a 16-fold and 7.5-fold increase in body exposure, an 11-fold and 26-fold increase in plasma half-life, respectively, and a dramatic reduction in LYC plasma clearance compared to free LYC. Although the best performance was obtained with PLA-PEG nanocapsules, PCL-nanocapsules also showed promising results. Finally, they reported a dose-dependent efficacy of LYC-PLA-PEG nanocapsules in mice infected with a *T. cruzi* strain resistant to benznidazole and nifurtimox (VL-10 strain) by the oral route, during both acute and chronic phases, whereas no cure was observed in animals treated with free LYC. These findings highlight the crucial role of nanotechnology in improving both the efficacy and biocompatibility of LYC.

Another SL-type compound, (−)-Cubebin (extracted from the dried seeds of *Piper cubeba* through fractionation processes), was encapsulated in PLGA microspheres using an emulsion/solvent evaporation method. In vivo assays in male BALB/c mice intraperitoneally infected with trypomastigotes of the *T. cruzi* Y strain showed that the encapsulated form significantly increased trypanocidal activity and prolonged survival compared to free (−)-Cubebin, confirming that biodegradable polymeric particles can enhance therapeutic outcomes of hydrophobic phytochemicals through controlled release and protection from degradation [195].

Similarly, the encapsulation of UA, a pentacyclic triterpene with well-known biological activity that is highly hydrophobic, in PCL-based NPs, was reported for the first time in 2015 for the treatment of acute *T. cruzi* infection in an animal model [196]. NPs were obtained by the nanoprecipitation method, and the selected formulation had a particle size of (173.2 ± 7.28) nm, low PDI (0.09), a zeta potential of (−36 ± 3.34) mV (important for the particle stability), and a high encapsulation efficiency of (94.1 ± 1.31)%. In vitro cytotoxicity assays demonstrated that UA-loaded NPs were non-toxic to fibroblast cells even at the highest concentrations studied, after 24 h or 48 h of incubation, and showed trypanocidal activity against *T. cruzi*. In vivo studies in mice infected with the Y strain of *T. cruzi* revealed a significant reduction in parasitemia comparable to benznidazole, but without the associated liver toxicity, highlighting the safety profile of the phytochemical-based nanocarrier system. This supports the biocompatibility advantage of PCL-based nanocarriers in delivering phytocompounds.

In a more integrative approach, curcumin, a polyphenolic compound derived from the rhizomes of *Curcuma longa*, recognized for its cardioprotective properties, particularly in the context of Chagas disease-associated cardiomyopathy, was co-administered in PLGA nanoparticles with a subtherapeutic dose of benznidazole. This combination markedly reduced cardiac parasite burden, hypertrophy, and inflammation in a chronic Chagas disease model, indicating that nanocarrier-enabled combination therapy can synergistically improve efficacy and reduce toxicity [197]. Curcumin-loaded NPs were prepared by a single emulsification process, followed by evaporation, and the dual regimen was superior to monotherapy in attenuating cardiac damage. These findings highlight the potential of nanocarrier-based combination therapies to enhance treatment efficacy while minimizing drug-related toxicity.

##### Nanoemulsions

Nanoemulsions have been explored as promising carriers for hydrophobic trypanocidal phytochemicals. In a recent study, a nanoemulsion formulation containing UA was developed using pseudoternary phase diagrams and hydrophilic–lipophilic balance techniques [198]. The nanoemulsion exhibited a small droplet size of 57.3 nm and a PDI of 0.24. The in vitro dissolution profile showed 75% release of UA after 5 min from the nanoemulsion in an alkaline dissolution medium, whereas only 20% was released from a physical mixture (components mixed without any emulsification) after 2 h. Biological evaluations using the CL Brener strain and LLC-MK2 monkey kidney fibroblasts confirmed that the nanoemulsion was effective against replicative forms of the parasite (amastigotes) and noncytotoxic. The calculated SI was 21.66, higher than 10, indicating that the system is a good candidate for in vivo assays.

Moreover, clove oil-based nanoemulsions have been used as vehicles and active ingredients, serving as the oil phase for sulfonamide encapsulation. These formulations have demonstrated potent antiprotozoan effects against two distinct *T. cruzi* strains, likely attributed to improved permeation of the enzyme inhibitor facilitated by the nanoemulsion system [199]. The dual role of essential oils (as bioactive agents and as nanocarrier components) represents a unique advantage of certain phytochemical-based nanoemulsions.

These findings highlight that nanoemulsions can offer key benefits for delivering poorly soluble compounds or enhancing the activity of repurposed drugs, depending on the formulation strategy. While polymeric nanoparticles often provide controlled release and long circulation times, nanoemulsions enable rapid release, improved solubilization, and enhanced intracellular delivery, making them suitable for compounds targeting intracellular parasites like *T. cruzi*.

##### Cyclodextrins

CD-based systems have also been explored to enhance the biopharmaceutical properties of plant-derived compounds with known trypanocidal activity. β-lapachone, a natural naphthoquinone (**44**), has demonstrated significant trypanocidal activity; however, its high cytotoxicity to mammalian cells and low aqueous solubility have limited its systemic administration and clinical application. To overcome these drawbacks, β-lapachone and its derivative nor-β-lapachone were complexed with 2-hydroxypropyl-β-cyclodextrin (2-HP-β-CD), achieving entrapment efficiencies above 80%. This strategy markedly improved the solubility of the compounds and promoted vectorization, enhancing their biological activity against both epimastigote and trypomastigote forms of *T. cruzi*, while also reducing cytotoxicity to mammalian cells. Notably, the inclusion complexes exhibited higher SIs compared to the free compounds, and even to benznidazole [200], highlighting the potential of CD-based systems for improving the therapeutic window of naphthoquinones.

In a subsequent study [201], the free and encapsulated forms of β-lapachone were further evaluated against the three evolutionary forms of *T. cruzi*, with a particular focus on amastigotes and trypomastigotes (the stages relevant to mammalian infection). Two strains were used: Tulahuen (DTU VI), which is susceptible to nitro derivatives, and Y (DTU II), which shows partial resistance to benznidazole. Encapsulation of β-lapachone significantly enhanced its activity against intracellular amastigotes of the Tulahuen strain in L929 fibroblasts, resulting in a 3.7-fold increase in efficacy. However, the improvement in selectivity was less pronounced due to a slight increase in the complex’s toxicity toward the fibroblast cell line. For the Y strain, no significant differences were observed between the free and complex forms of β-lapachone in terms of activity against amastigotes in cardiac cells. This indicates that while encapsulation enhanced solubility and selectivity in some contexts, it did not alter the trypanocidal activity of the compound in all cases.

These findings illustrate the context-dependent advantages of CD complexes: while they enhance solubility and bioactivity, their overall performance in terms of selectivity and strain coverage can vary. Compared to other nanosystems such as nanoemulsions or polymeric NPs, CDs offer simple preparation and high drug loading but may be less versatile in modulating pharmacokinetics or bypassing resistance in certain strains.

**Table 2:** Summary of nanocarrier systems incorporating antichagasic phytochemicals.

Nanocarrier	Nanocarrier Composition	Plant Name	Main Chemical Constituent/s	Size (nm)	Stage of Development	Reference
Inorganic NPs	AgNPs	Corn Cobs	Xylan (D-xylose, L-arabinose, 4-O-methyl-D-glucuronic acid)	40–55.3	In vitro (epimastigote forms of the Y strain of *T. cruzi*)	[189]
Polymeric nanocapsules	PCL	*Lychnophora* *trichocarpha*	LYC	190.2	Preclinical (Swiss mice)	[190]
				175–245	Preclinical (Female Swiss albino mice)	[194]
	PLA-PEG			106.1	Preclinical (Swiss mice)	[190]
				105.1	Preclinical (C57BL/6 mice)	[192]
				105–138	Preclinical (Female Swiss albino mice)	[194]
				107	Preclinical (Female Swiss albino mice)	[193]
Polymeric particles	PLGA	*Piper cubeba*	(−)-Cubebin	1000	Preclinical (BALB/c mice)	[140]
	PCL-Poloxamer 407	NA	UA	173.2	Preclinical (Swiss mice)	[195]
	PLGA-PVA	*Curcuma longa*	Curcumin	250–300	Preclinical (C57BL/6 mice)	[197]
Liposomes	DPPC	*Cortex Frangula*	*Hypericum* *perforatum*	NA	Trypomastigote forms of the *T. cruzi* Y strain	[202]
Micelles	Pluronic™ F-127					
	Pluronic™ F-123					
Nanoemulsion	Capryol^®^ 90, Cremophor^®^ EL/Transcutol^®^ P	NA	UA	57.3	In vitro (amastigote forms of the clone CL Brener strain B5 of *T. cruzi*)	[198]
	Pluronic F127^®^	*Eugenia caryophyllus* and other trypanocidal agent (sulfonamides)	Essential oils (eugenol)	35–100	In vitro (Epimastigote forms of the *T. cruzi* clone Dm28c (lineage TCII)31 and Y (lineage TCI))	[199]
CDs	2-hydroxypropyl-β-cyclodextrin	NA	β-lapachone (naphthoquinone)	NA	In vitro trypomastigotes of the Tulahuen strain, intracellular amastigote, epimastigotes, and trypomastigotes (Y strain)	[201]
	2-hydroxypropyl-β-cyclodextrin	NA	β-lapachone and nor-β-lapachone	NA	Epimastigote and trypomastigote forms of *T. cruzi* (strain Y)	[200]

NA: not available.

## 5. Remarks and Future Perspectives

The persistent burden of neglected parasitic diseases such as Chagas disease and malaria continues to pose significant global health challenges, particularly in low- and middle-income countries. Despite the long-standing recognition of phytochemicals as effective antiparasitic agents, their full therapeutic potential remains underexploited due to limitations related to bioavailability, stability, and targeted delivery. The integration of nanotechnology with phytochemistry, herein referred to as phytonanotechnology, represents a promising paradigm shift capable of overcoming these challenges.

This review underscores the growing body of evidence supporting the efficacy of PNOs in combating neglected parasitic infections. When formulated into nanocarriers, these bioactivities can exhibit improved pharmacokinetic and pharmacodynamic profiles, leading to enhanced therapeutic outcomes with potentially reduced toxicity and side effects. In particular, phytonanoformulations can enable targeted delivery, controlled release, and improved cellular uptake, offering novel avenues for the treatment of parasitic infections that have long been underserved by conventional pharmaceutical strategies.

However, despite encouraging in vitro and in vivo results, the clinical translation of phytonanoformulations remains limited. A major barrier is the lack of standardized protocols for the reproducible synthesis, characterization, and evaluation of these nanosystems. Moreover, the quality control and scalability of PNO-loaded nanocarriers, especially those based on complex plant extracts, are hindered by batch-to-batch variability and difficulties in sustainably sourcing bioactive products. There is a pressing need for rigorous toxicological assessments, long-term stability studies, and cost-effectiveness analyses to validate the feasibility of large-scale production and implementation in endemic regions. Regulatory barriers further complicate the path to clinical application, as existing frameworks often lack clear guidelines for complex plant-based nanotechnological products. Additionally, comprehensive in vivo studies, including toxicological evaluations, pharmacokinetic analyses, and biodistribution profiling, are still lacking for many proposed systems.

Looking ahead, future research should prioritize: (a) the standardization of methodologies for the development and quality control of phytonanoformulations; (b) mechanistic studies to better understand the interactions among PNOs, nanocarriers, and parasitic targets; (c) in vivo and clinical investigations to evaluate therapeutic efficacy, safety, and pharmacokinetics; (d) scalable and sustainable sourcing methods, including green synthesis approaches for both phytochemicals and nanomaterials to ensure environmental and economic viability; (e) interdisciplinary collaborations between natural product chemists, nanotechnologists, parasitologists, pharmacists and public health experts to accelerate translational pathways and foster regulatory frameworks tailored to the unique challenges of plant-based nanomedicines.

Multidisciplinary collaboration will be essential to overcoming these barriers. By integrating expertise from natural product chemistry, nanotechnology, pharmacology, parasitology, and regulatory science, it is possible to accelerate the development of effective and accessible phytonanotechnological therapies for neglected parasitic diseases.

In conclusion, although phytonanotechnology is still an emerging field, it holds transformative potential in the fight against neglected parasitic infections. By bridging traditional plant-based medicine with cutting-edge nanoscience, this approach offers a pathway toward safer, more effective, and accessible treatments. Addressing current limitations and regulatory challenges will be crucial to unlocking its full potential in the global fight against parasitic infections.

## Data Availability

No new data were created or analyzed in this study. Data sharing is not applicable to this article.

## Abbreviation

ART: artemisinin; CDs: cyclodextrins; CPT: complete protection time; DEET: N,N-diethyl-meta-toluamide; DMPEmPEG2000: dimyristoylphosphatidylethanolamine-polyethylene glycol 2000; DOPC: 1,2-dioleoyl-*sn*-glycero-3-phosphocholine; DOTAP: *N*-[1-(2,3-dioleoyloxy)propyl]-N,N,N-trimethylammonium methyl-sulfate; IFN-γ: interferon gamma; IL-10: interleukin-10; IC_50_: half-maximal inhibitory concentration; LYC: lychnopholide; LC_50_: median lethal concentration; MCT: caprylic/capric triglyceride oil; MESBMA: morpholinoethyl sulfobetaine methacrylate; mPEG: methoxypoly (ethylene glycol); NLCs: nanostructured lipid carriers; NPs: nanoparticles; PBMA: poly(butyl methacrylate); PCL: poly-caprolactone; PDI: polidispersity index; PEG: polyethylene glycol; PLA: poly(D,L-lactide); PLGA: poly (lactic-co-glycolic acid); PNOs: products of natural origin; PVA: polyvinyl alcohol; P80: polysorbate 80; SI: selectivity index; SAR: structure–activity relationship; SLNs: solid lipid nanoparticles; SLs: sesquiterpenic lactones; SMEDDS: self-microemulsifying drug delivery systems; Span 60: sorbitan monostearate; S75: soy phosphatidylcholine; TEM: transmission electron microscopy; TNF: tumor necrosis factor; UA: ursolic acid.
